# Supplementary material for: Spin-valley locking and bulk quantum Hall effect in a noncentrosymmetric Dirac semimetal BaMnSb2
Source: Nat Commun. 2021 Jul 1;12:4062. doi: 10.1038/s41467-021-24369-1 (PMC8249485; doi:10.1038/s41467-021-24369-1)
Supplement: Supplementary file 1 — Supplementary Information [file 41467_2021_24369_MOESM1_ESM.pdf]

Supplementary Information
Spin-valley locking and bulk quantum Hall effect in a noncentrosymmetric Dirac semimetal $BaMnSb_2$

CONTENTS

I. Supplementary Notes 7
1. Orthorhombic lattice distortion detected by neutron scattering 7
2. SHG polarimetry and Theoretical Modeling 7
3. 2D electronic structure of $BaMnSb_2$ 8
4. Tunneling mechanism for interlayer transport 9
5. Transport measurements on sample $E\#1$ 10
6. Zeeman Effect of $BaMnSb_2$ 11
7. Possible origins of the z-axis resistance plateau in the quantum Hall state 12

II. Supplementary Discussions 13

References 25

Lattice constants (Å)	a=4.5036	b=4.4701	c=24.6120
Wyckoff positions	x	y	z
Ba:4d	0.0059	0	0.1135
Mn:4d	0.5046	0	0.2501
Sb:2a	0.5172	0	0
Sb:2b	0.9511	0.5	0
Sb:4d	0.5054	0.5	0.1853

Supplementary Table 1. Lattice constants and Wyckoff positions of $BaMnSb_2$ with the space group I2mm derived from the DFT calculations.

**Sample label**	**Nominal composition**	μ_c **(cm^2/V s)**	$n_{\rm Hall}$ **(10^{19} cm^{-3})**	F **(T)**	n_{2D} **(10^{12}cm^{-2})**	$n_{\rm SdH}$ **(10^{19} cm^{-3})**	Φ_B	**s**
B#1	$BaMnSb_2$	1645	0.61	18.83	0.91	0.76	0.97π	1.5
E#1	$Eu_{0.1}Ba_{0.9}MnSb_2$	5040	1.4	30.58	1.48	1.24	0.96π	2.3
E#2	$Eu_{0.1}Ba_{0.9}MnSb_2$	-	-	34.40	1.65	1.38	0.89π	-
E#3	$Eu_{0.1}Ba_{0.9}MnSb_2$	556	0.74	17.90	0.87	0.72	0.91π	2.2
Z#1	$BaMn_{0.9}Zn_{0.1}Sb_2$	580	0.093	8.9	0.43	0.36	0.86π	1.5

Supplementary Table 2. **Quantum transport parameters of the five samples in the context.** μ_c, the classical carrier mobility, and $n_{\rm Hall}$, the carrier density, extracted from the Hall measurement. F is the quantum oscillation frequency. n_{2D} is the 2D carrier density in each Sb layer, derived by $n_{2D} = 2eF/h$ according to Luttinger's theorem, where h is Planck's constant. The 3D carrier density is derived using $n_{\rm SdH} = n_{2D}/(c/2)$, where c is the lattice parameter. Φ_B is the Berry phase and s is the degeneracy per Sb layer.

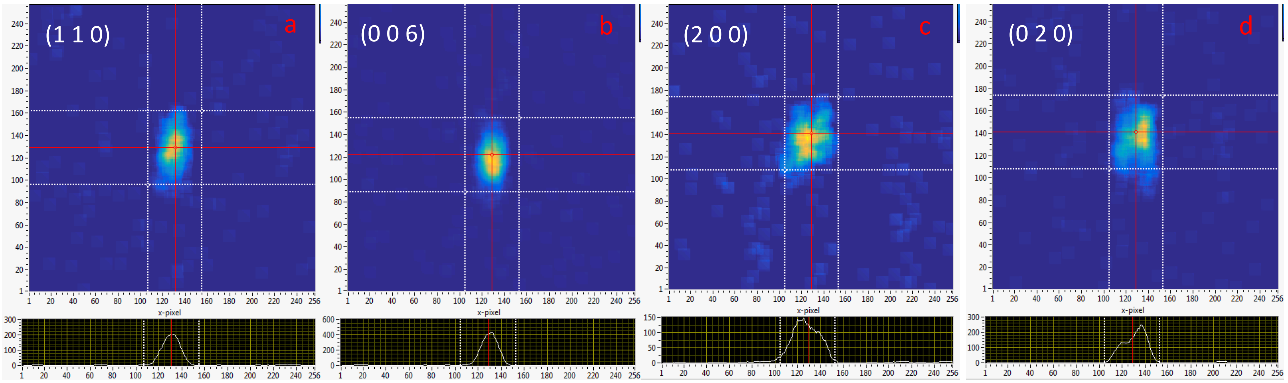

Supplementary Figure 1. **a-d** show the observed single crystal neutron diffraction spots at the detector at the Bragg peak positions of (1 1 0), (0 0 6), (2 0 0), and (0 2 0), respectively, T = 300K. The detector pixels (256 x 256) are showed for each plot. The bottom part of each plot shows the integrated intensity over vertical pixels in the range between two white dotted lines. The peak region framed by the whited dotted lines has the same size for each peak spot as guide for eyes to tell the peak height and width. One can notice the peak spots (2 0 0) and (0 2 0) are broader than that of (1 1 0) and (0 0 6) in the horizontal width, indicating that an orthorhombic lattice distortion exists in $BaMnSb_2$.

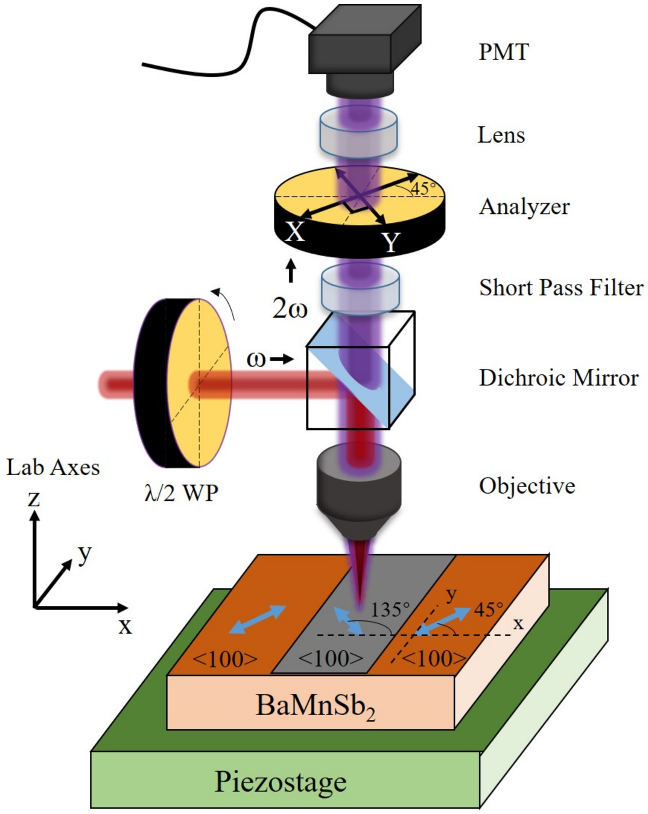

Supplementary Figure 2. Schematic of the SHG microscopy setup. For SHG imaging, images of (001) - cleaved $BaMnSb_2$ were obtained at a resolution of 3 points per micrometer by moving piezostage. For polarimetry measurement, the polarization angle (θ) of the incident laser was continuously tuned from 0° to 360° by rotating the $\lambda/2$ wave plate.

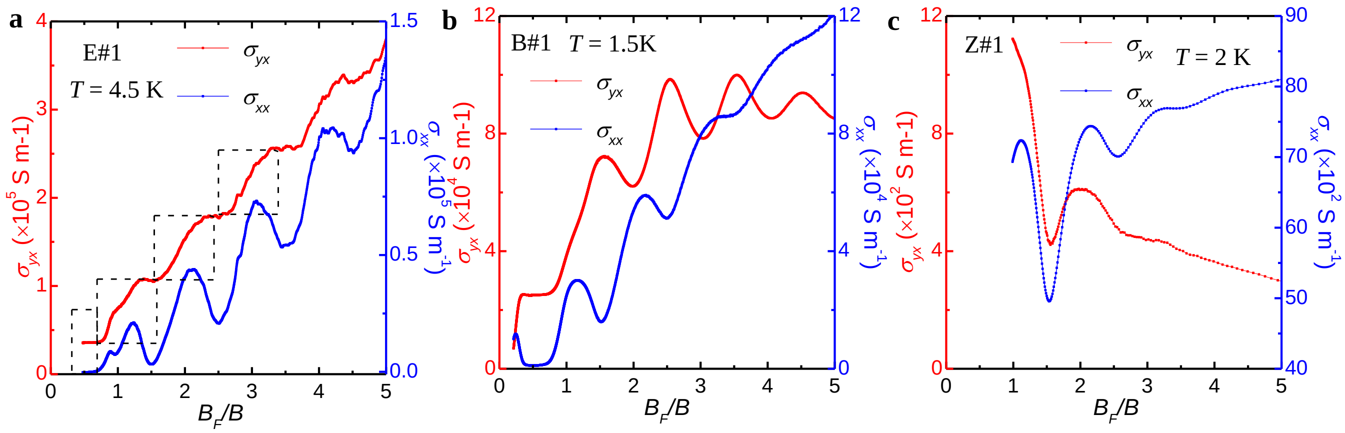

Supplementary Figure 3. Hall conductivity σ_{yx} and longitudinal conductivity σ_{xx} for samples E#1 (a), B#1 (b) and Z#1 (c). Both σ_{yx} and σ_{xx} are obtained from tensor conversions from ρ_{xx} and ρ_{xy}.

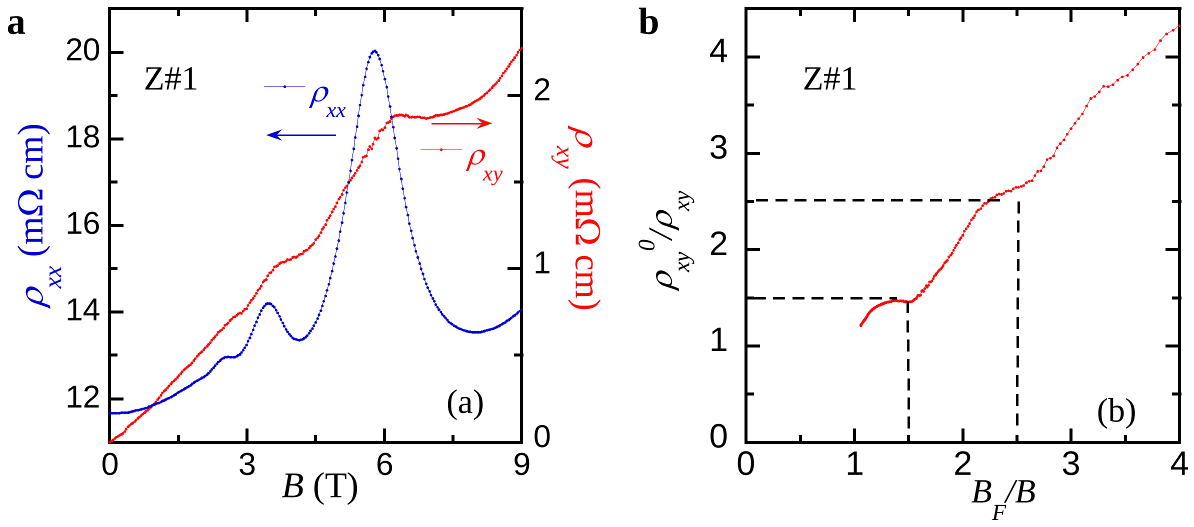

Supplementary Figure 4. QHE of sample Z#1. **a**, Magnetic field dependence of in-plane (ρ_{xx}) and Hall (ρ_{xy}) resistivity up to 9T at 2K. **b**, Normalized inverse Hall resistivity (ρ^0_{xy}/ρ_{xy}) at 2K

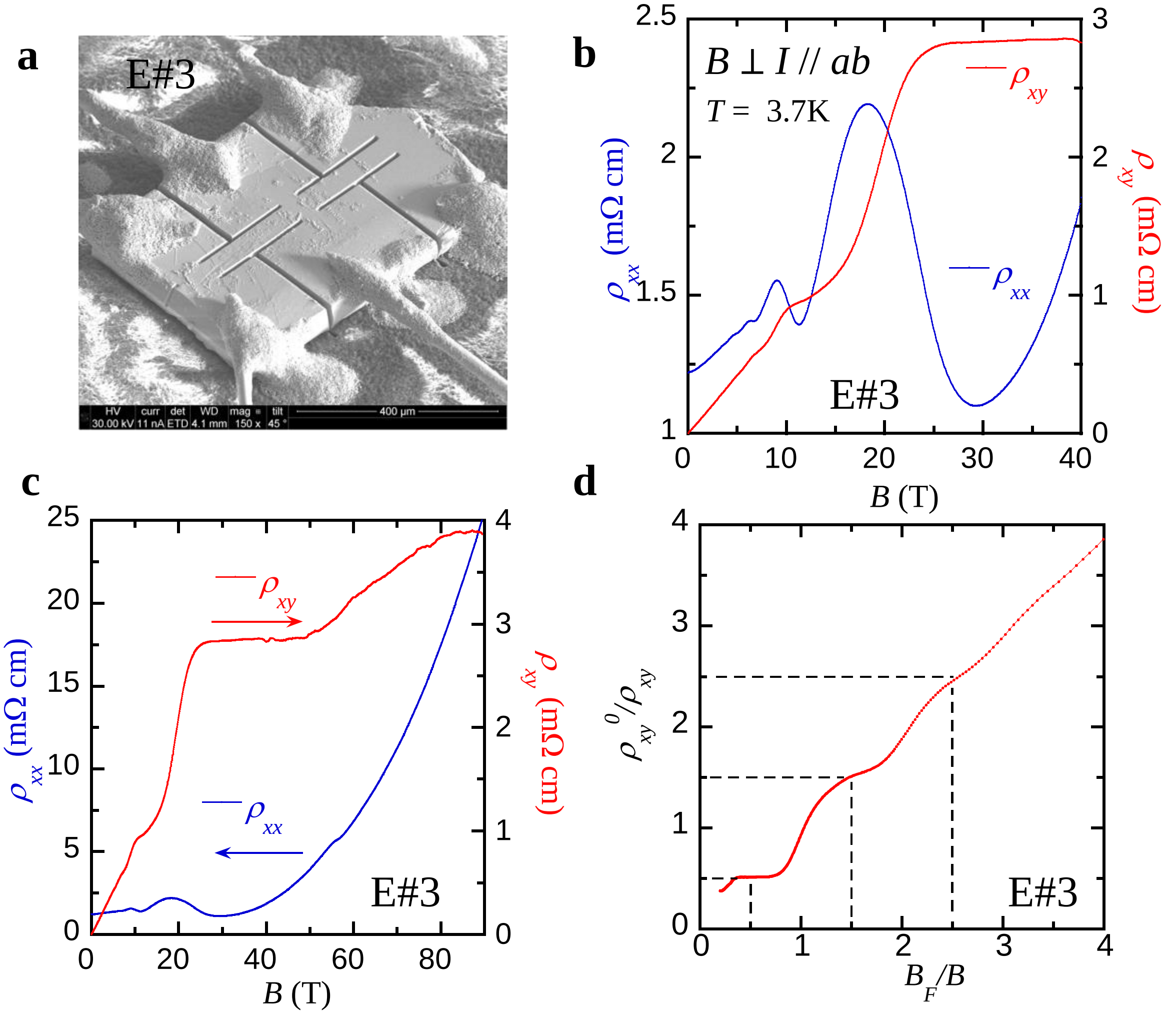

Supplementary Figure 5. QHE of sample E#3. **a**, SEM image of the Hall-bar sample E#3 fabricated by FIB. **b**, Magnetic field dependence of in-plane (ρ_{xx}) and Hall (ρ_{xy}) resistivity up to 40T at 3.7 K. **c**, ρ_{xx} and ρ_{xy} vs. magnetic field up to 90 T at 3.7K. **d**, Normalized inverse Hall resistivity (ρ^0_{xy}/ρ_{xy}) at 3.7K.

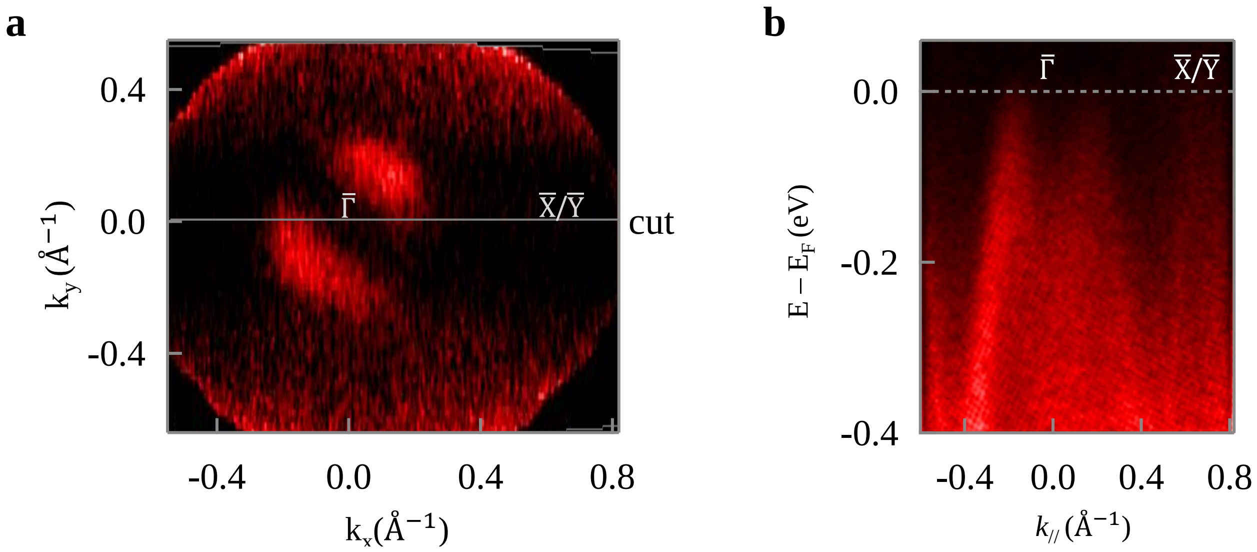

Supplementary Figure 6. a, Constant energy contour of $Ba(Mn_{0.9}Zn_{0.1})Sb_2$ on the $k_x - k_y$ plane, which is acquired by integrating from -15 meV to -5 meV. The ARPES data is taken with the photon energy of 25 eV. b, ARPES spectrum along the cut. There is a hole pocket crossing the Fermi level around the $\bar{\Gamma}$ point.

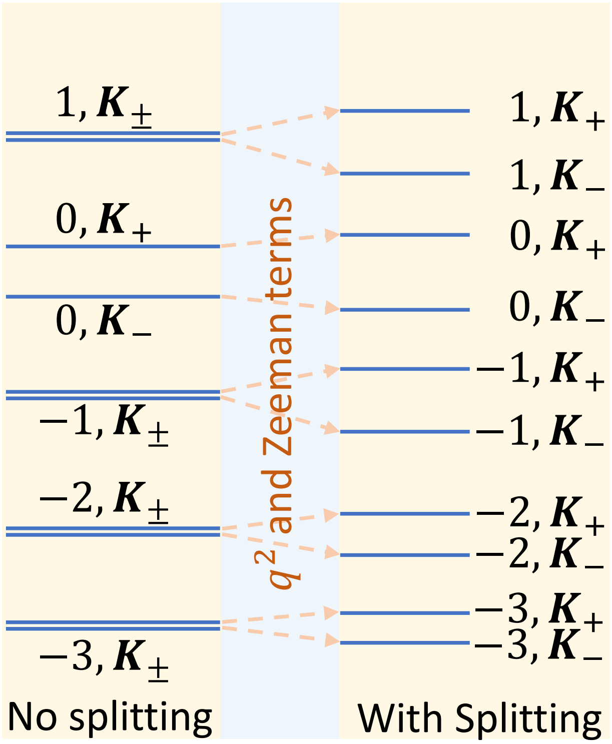

Supplementary Figure 7. Schematic illustration of the valley splitting due to the $\boldsymbol{q}$-quadratic and Zeeman terms.

I. SUPPLEMENTARY NOTES

1. Orthorhombic lattice distortion detected by neutron scattering

As mentioned in the main text, the crystal structure of $BaMnSb_2$ were reported to be tetragonal with a space group of $I4/mmm$ in several studies [1–4]. In our previous work, we did neutron scattering measurements to determine the magnetic structure of $BaMnSb_2$ [1]. Based on 164 nuclear reflection peaks, the refinement of the crystal structure showed a roughly good fit using the tetragonal structural model. However, after a careful inspection of individual reflection peaks, we did find broadening in the diffraction spots at (200) and (020). The integrated intensity for (020) show noticeable peak splitting, as shown in supplementary Fig. 1. This suggests the real structure of $BaMnSb_2$ should involve an orthorhombic lattice distortion, which is further clarified by our STEM study (see text).

2. SHG polarimetry and Theoretical Modeling

SHG is a nonlinear optical process only shown in materials with non-centrosymmetric point groups, which is widely used in crystal symmetry study. In the process, light with a frequency of ω is incident on a crystal, inducing an electromagnetic polarization with a frequency of 2ω. [5] The induced polarization $P^{2\omega}$ can be expressed as,

$$P_i^{2\omega} = \varepsilon_0 \chi_{\mathrm{ijk}} E_j^{\omega} E_k^{\omega}$$

where χ_{ijk} is known as the second nonlinear optical susceptibility.

In Voigt notation, χ_{ijk} can be simplified to a 3×6 matrix d_{ij} (i=1,2,3; l=1,2,3,4,5,6), where the index l=1,2,3,4,5,6 in d_{ij} corresponds to jk=11,22,33,23/32,13/31,12/21 in χ_{ijk} respectively. Therefore, polarization $P^{2\omega}$ can be written as,

$$\begin{pmatrix} P_1^{2\omega} \\ P_2^{2\omega} \\ P_3^{2\omega} \end{pmatrix} = \varepsilon_0 \begin{pmatrix} d_{11} & d_{12} & d_{13} & d_{14} & d_{15} & d_{16} \\ d_{21} & d_{22} & d_{23} & d_{24} & d_{25} & d_{26} \\ d_{31} & d_{32} & d_{33} & d_{34} & d_{35} & d_{36} \end{pmatrix} \begin{pmatrix} (E_1^{\omega})^2 \\ (E_2^{\omega})^2 \\ (E_3^{\omega})^2 \\ 2E_2^{\omega}E_3^{\omega} \\ 2E_3^{\omega}E_1^{\omega} \\ 2E_1^{\omega}E_2^{\omega} \end{pmatrix}$$

From Neumann's principle, the SHG d matrix for the $2mm$ symmetry, point group with 2-fold axis along with crystal a-axis, is

$$d_{\mathrm{ij}} = \begin{pmatrix} d_{11} & d_{12} & d_{13} & 0 & 0 & 0 \\ 0 & 0 & 0 & 0 & 0 & d_{26} \\ 0 & 0 & 0 & 0 & d_{35} & 0 \end{pmatrix}$$

where polar in-plane a-axis is along direction given by subscript 1, in-plane b axis is along direction 2, and out-of-plane c-axis is 3. If instead, the polar a-axis is along the direction 2, then the d_{ij} will transform to

$$d'_{\mathrm{ij}} = \begin{pmatrix} 0 & 0 & 0 & 0 & 0 & d_{26} \\ d_{12} & d_{11} & d_{13} & 0 & 0 & 0 \\ 0 & 0 & 0 & d_{35} & 0 & 0 \end{pmatrix}$$

For our experimental set up with normal incidence, $E_1^{\omega} = E\cos\theta$, $E_2^{\omega} = E\sin\theta$, $E_2^{\omega} = 0$. For simplicity, set E=1, then the SHG signal from each domain can be described by:

$$\begin{cases} I_X^{2\omega} = \left(P_X^{2\omega}\right)^2 = \left(P_1^{2\omega}\right)^2 = |d_{11}|^2\cos^4\theta + |d_{12}|^2\sin^4\theta + |d_{11}|\,|d_{12}|\cos^2\theta\sin^2\theta \\ I_Y^{2\omega} = \left(P_Y^{2\omega}\right)^2 = \left(P_2^{2\omega}\right)^2 = |d_{26}|^2\sin^2(2\theta) \end{cases}$$

$$\begin{cases} I_X^{2\omega'} = \left(P_X^{2\omega'}\right)^2 = \left(P_1^{2\omega'}\right)^2 = |d_{26}|^2\sin^2(2\theta) \\ I_Y^{2\omega'} = \left(P_Y^{2\omega'}\right)^2 = \left(P_2^{2\omega'}\right)^2 = |d_{12}|^2\cos^4\theta + |d_{11}|^2\sin^4\theta + |d_{11}|\,|d_{12}|\cos^2\theta\sin^2\theta \end{cases}$$

In our modeling of the twin domains in the experimental images shown in Figure 1, a simple single domain with tensor d_{ij}, and another adjacent twin domain with a tensor d_{ij}' does not provide the best fit to the polarimetry data shown in Figure 1. Since the domains are large (microns) relative to the beam size (~180nm), the polarimetry should have fit with single domain expressions given above. [6] Since they did not, we had to assume the presence of twin domains underneath the surface domains as follows:

$$\begin{cases} I_X^{2\omega}\,(\text{total}) = I_X^{2\omega} + k_1 \cdot I_X^{2\omega'} \\ I_Y^{2\omega}\,(\text{total}) = I_Y^{2\omega} + k_1 \cdot I_Y^{2\omega'} \end{cases}$$

$$\begin{cases} I_X^{2\omega}\,(\text{total})' = I_X^{2\omega'} + k_2 \cdot I_X^{2\omega} \\ I_Y^{2\omega}\,(\text{total})' = I_Y^{2\omega'} + k_2 \cdot I_Y^{2\omega} \end{cases}$$

where k_1 and k_2 is a coefficient described the proportion of intensity comes from the hidden domains. Such domains beneath the surface in a polar metal was also seen recently in another polar metal, $Ca_3Ru_2O_7$. [7] The equations above provided good fits to our experimental polarimetry data in Figure 1. Future cross-sectional TEM study of the exact area that was optically probed should provide a confirmation of the presence of such domains.

3. 2D electronic structure of $BaMnSb_2$

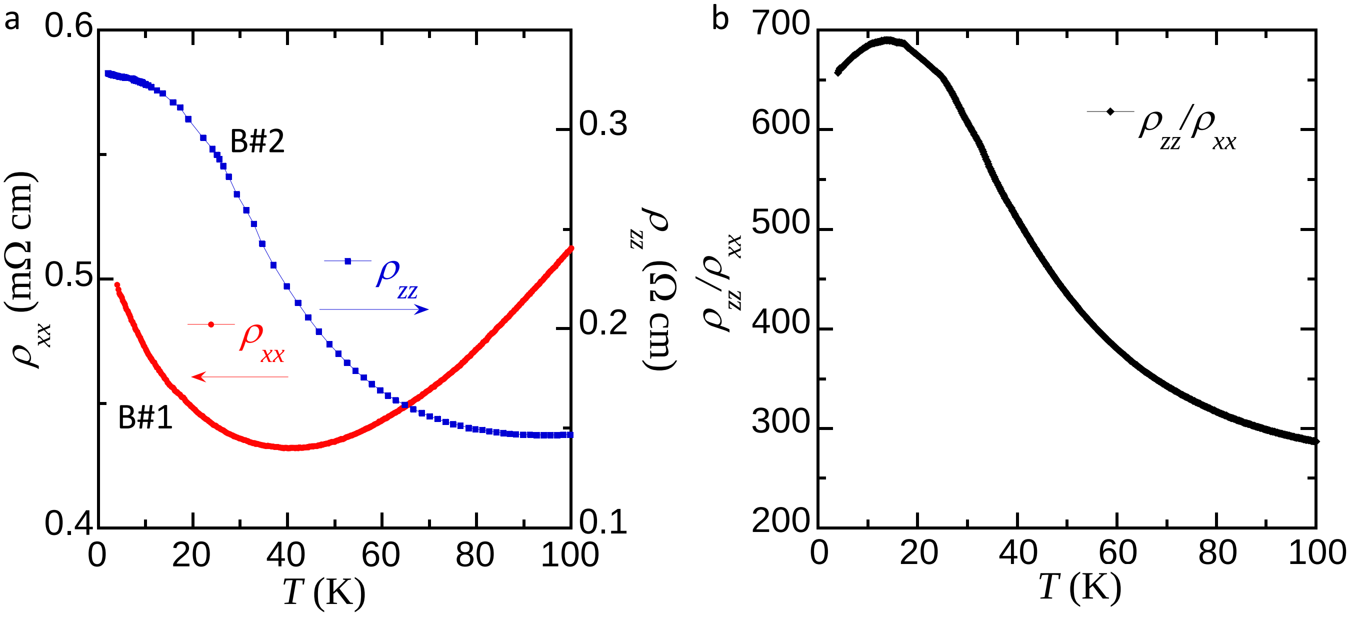

Supplementary Figure 8. Large anisotropic ratio in $BaMnSb_2$. (**a**) Temperature dependence of in-plane (ρ_{xx}) and out-of-plane (ρ_{xy}) resistivity. (**b**) Temperature dependence of anisotropic ratio ρ_{zz}/ρ_{xx}.

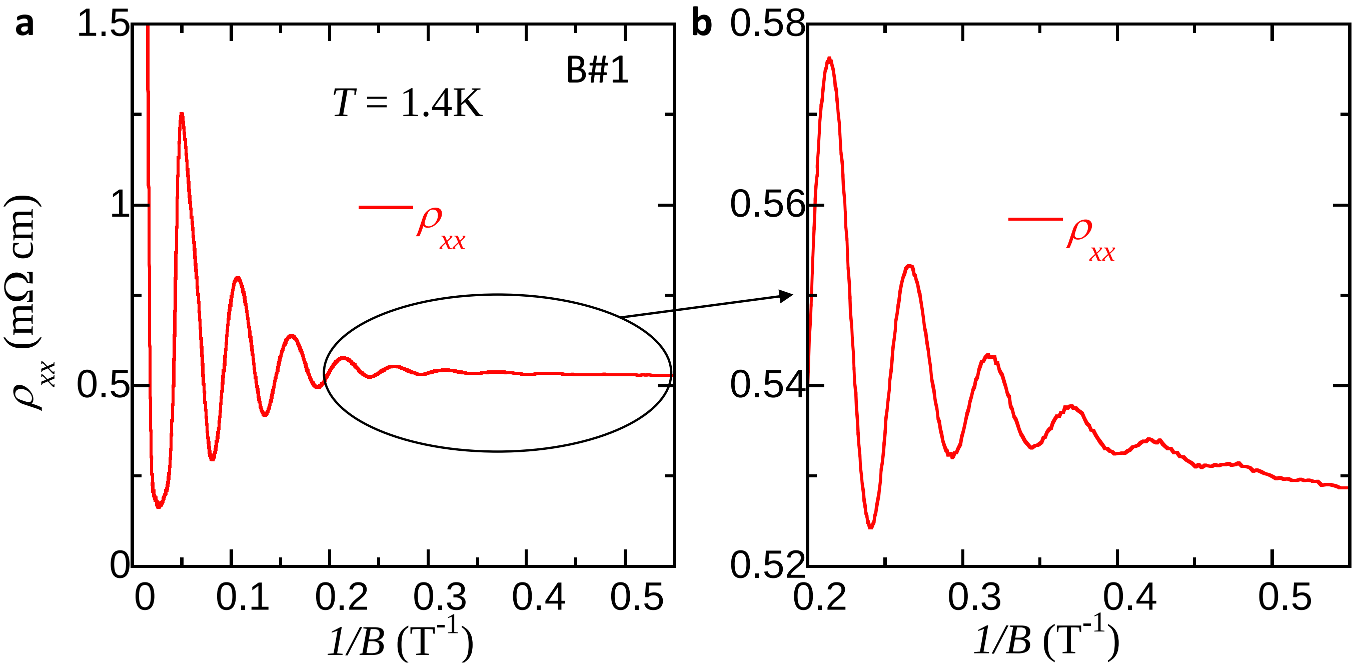

Supplementary Figure 9. (**a**) In-plane resistivity ρ_{xx} versus *1/B* for samples B#1 and (**b**) Zoom-in view for the low field range (0.2 T^{-1} < 1/B <0.55 T^{-1}) in (**a**).

The small k_z-dispersion in electronic band structure (Fig. 2a, main text) suggests a quasi-2D electronic state in $BaMnSb_2$. This is further supported by the quantum oscillation studies. A perfectly 2D electronic state is characterized by straight cylindrical Fermi surface, while in a layered material with large anisotropic ratio ρ_{zz}/ρ_{xx}, which is the case for $BaMnSb_2$ (supplementary Fig. 8), a quasi-2D state with corrugated cylindrical Fermi surface is expected. With magnetic field being applied perpendicular to the plane direction (i.e., along the FS cylinder), there exist both minimum and maximum FS cross-sections perpendicular to magnetic field, leading to multiple frequencies in quantum oscillations. Given these frequencies are not far from each other, the interference of these oscillation components leads to oscillation beating like pattern, which has been observed in quantum oscillation of ρ_{xx} in sample B#1. As shown in supplementary Fig. 9, the oscillating amplitude continuously increases as the $1/B$ decreases in the range of [0.05T^{-1}, 0.5T^{-1}], which indicates the beating frequency is

$$F_{\text{beating}} < 1/[2 \times (0.5 - 0.05)] = 1.1\text{T} \ . \tag{1}$$

Assuming there are two frequency components (F_1 and F_2) due to FS corrugation, the frequency difference can be estimated by:

$$\Delta F = F_1 - F_2 = F_{\text{beating}} < 1.1\text{T} \ . \tag{2}$$

Compared with the average oscillation frequency of 18.83T obtained from the FFT of the oscillation pattern, the relative corrugation is as small as $\Delta F/F < 6\%$. This indicates that FS is nearly a straight cylinder, demonstrating the nearly prefect 2D electronic structure of $BaMnSb_2$.

4. Tunneling mechanism for interlayer transport

Institutively, the large anisotropic ratio ρ_{zz}/ρ_{xx} (supplementary Fig. 8) implies a tunneling mechanism for interlayer (out-of-plane) transport in layered $BaMnSb_2$. More solid evidence for interlayer tunneling can be found in the in-phase oscillations of in-plane (σ_{xx}) and out-of-plane(σ_{zz}) conductivities. supplementary Fig. 10 presents the field dependence of conductivity for samples B#2 and E#2. σ_{xx} was obtained from the 2D resistivity tensor inversion, while σ_{zz} should be derived from the 3D resistivity tensor inversion [8]:

$$\sigma_{zz} = \frac{\rho_{xx}\rho_{yy} - \rho_{xy}\rho_{yx}}{\rho_{xx}\rho_{yy}\rho_{zz} - \rho_{xy}\rho_{yx}\rho_{zz} - \rho_{xz}\rho_{zx}\rho_{yy}} \ . \tag{3}$$

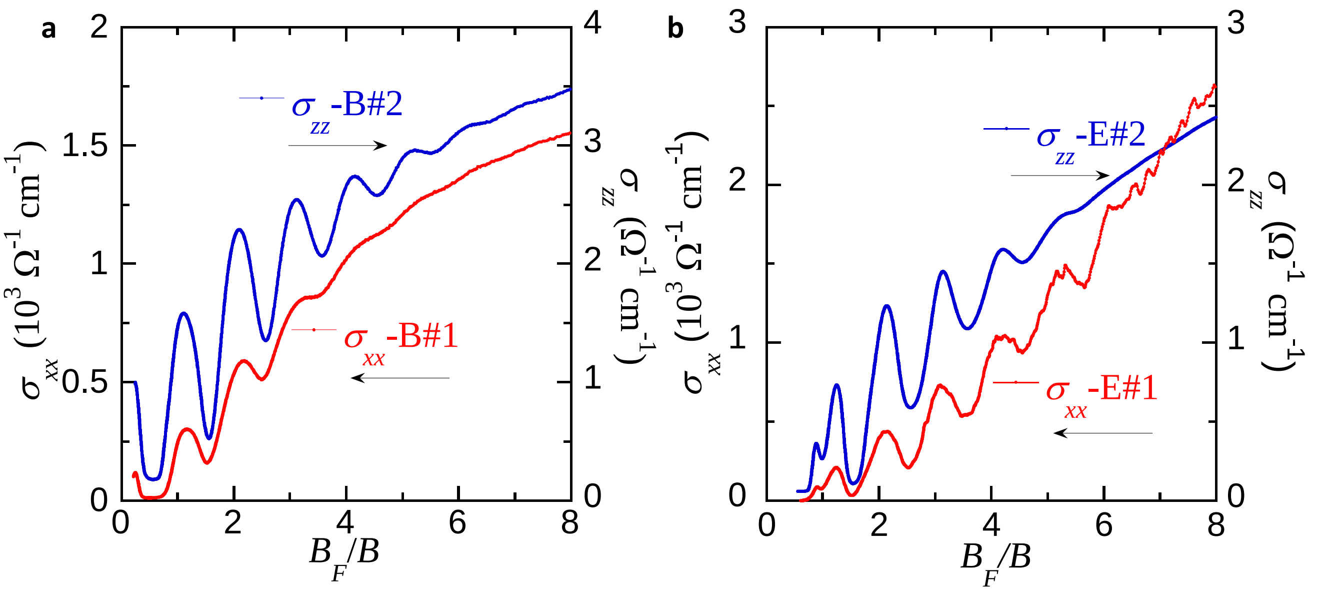

Supplementary Figure 10. In-plane (σ_{xx}) and out-of-plane (σ_{zz}) conductivities for samples B#1 and B#2 (**a**) and samples E#1 and E#2 (**b**).

Given $\rho_{zz} \gg \rho_{xx} = \rho_{yy}$, and $\rho_{xz} = \rho_{yz} = 0$ when magnetic field is along the z-axis, the above equation approximates to $\sigma_{zz} \approx 1/\rho_{zz}$.

In 3D QHE, the in-plane transport exhibits QHE behavior but the out-of-plane transport does not. For the in-plane transport, the nearly zero σ_{xx} plateau at high fields is indeed the signature of non-local transport of QHE, which corresponds to the situation that the Fermi level (E_F) resides in between Landau levels (LLs) (i.e., DOS(E_F) achieves a minimum).

For the out-of-plane transport, if the transport is due to the momentum relaxation (i.e., non-tunneling), σ_{zz} should reach a maximum at a minimum DOS(E_F) according to the quantum oscillation theory [9–12]. The earlier transport theory has established that the scattering probability ($1/\tau$) is proportional to the number of available states that electron can be scattered into, so $1/\tau$ oscillates in concert with the oscillations of DOS(E_F)[9–12]. Hence, $\sigma \propto \tau \propto 1/\text{DOS}(E_F)$, which has been experimentally observed [13]. This is inconsistent with the observation that σ_{zz} reaches a minimum at the σ_{xx} plateau (i.e., at DOS(E_F) minimum, supplementary Fig. 10a and 10b). On the other hand, the tunneling current should be proportional to DOS(E_F), so the tunneling conductivity $\sigma \propto \text{DOS}(E_F)$. This is exactly what have been observed. In the quantum Hall state within the quantum limit, the gap between Landau levels is significantly increased, which minimizes the interlayer tunneling. In this case, the z-axis transport is dominated by the 2D chiral surface state, which is manifested by the saturation tendency of ρ_{zz} below 20K (Fig. 4c in the main text),

5. Transport measurements on sample E#1

The E#1 Hall-bar sample was also prepared through focused ion beam (FIB) cutting. supplementary Fig. 11a shows the optical image of this sample. During cooling-down for pulse field measurements on this sample, leads #2 and 4 broke, so we had to use leads #3 and #6 to measure both ρ_{xy} and ρ_{xx}. supplementary Fig. 11b presents the raw data of the voltage measured between leads #3 and #6, $V_{3,6}$. Although leads #3 and #6 are significantly misaligned, $V_{3,6}$ show a remarkable asymmetric feature between positive and negative magnetic fields, indicating $V_{3,6}$ is dominated by the Hall voltage V_{xy} and the longitudinal voltage V_{xx} is small. supplementary Fig. 11c shows V_{xx} and V_{xy} data obtained through symmetrizing and anti-symmetrizing of the $V_{3,6}$ data acquired under positive and negative magnetic fields. The V_{xy} plateaus are found to be accompanied by the V_{xx} minima, a typical signature of QHE. The small negative V_{xx} above 47.5T can be attributed to the fact that symmetrizing $V_{3,6}$ between positive and negative fields cannot completely remove the Hall voltage component, which is often seen in Hall effect measurements where the longitudinal and Hall resistivities are mixed. The anti-symmetrizing process of $V_{3,6}$ may also not completely remove V_{xx} from V_{xy}, but the perfect V_{xy} plateau near 50T (supplementary Fig. 11c) indicates the ρ_{xx} at this quantum Hall state is extremely small. The longitudinal conductivity σ_{xx} for this quantum Hall state is indeed close to zero, as shown in Fig. 4d in the main text. The observations of σ_{yx} equal steps in sample E#1 (Fig. S3a), together with its

very small ρ_{xx} (Fig. 4a) and nearly zero σ_{xx} at the quantum Hall state within the quantum limit (Fig. 4d), suggests its stacked QHE is nearly perfect; that is, almost every 2D Sb conducting layer acts as a quantum Hall layer.

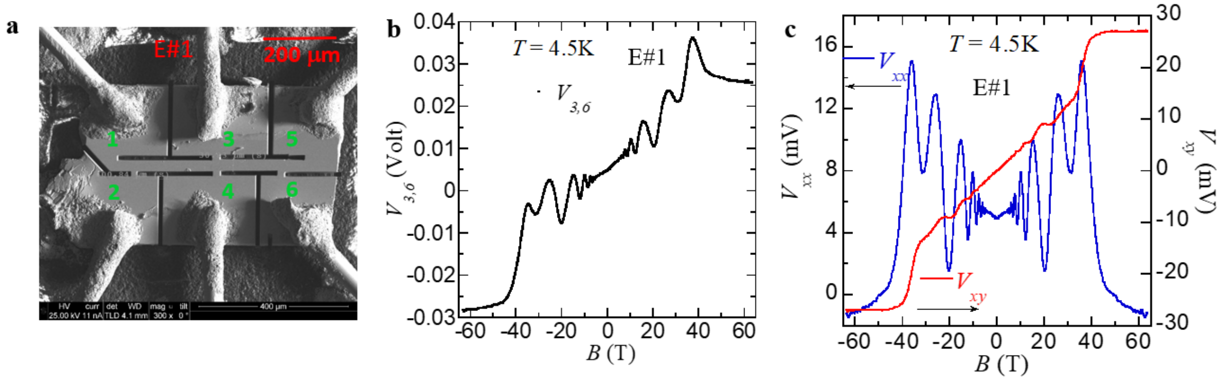

Supplementary Figure 11. **a**, The SEM image of the Eu-doped $BaMnSb_2$ sample (E#1). **b**, The field dependence of the voltage between leads 3 and 6 ($V_{3,6}$) for sample E#1 at 4.5K. **c**, The Hall voltage V_{xy} and longitudinal voltage V_{xx} obtained via anti-symmetrizing and symmetrizing the $V_{3,6}$ data respectively.

6. Zeeman Effect of $BaMnSb_2$

To evaluate the Zeeman effect in $BaMnSb_2$, we have recently measured the angular dependence of the Shubnikov-de Haas (SdH) oscillations in interlayer resistance R_{ZZ} for a Eu-doped sample (supplementary Fig. 12a). For such measurements, if the g-factor of the material is large, we would expect to observe the SdH oscillations are modulated by the tilt angle θ of magnetic field: the oscillation amplitude varies with θ and decreases to zero at a critical angle θ_c, above which the SdH oscillations exhibits a phase inversion. This is an approach often used to determine the g-factor for layered materials and known as the spin-zero method [14]. For instance, this method was recently used to determine the g-factor of $EuMnBi_2$ [15]. The critical angle θ_c was found be $\sim 40°$ for $EuMnBi_2$ and the g-factor estimated using the relation $\cos\theta_c = gm_c/m_0$ (m_c, cyclotron mass; m_0, free electron mass) is 9.8(4).

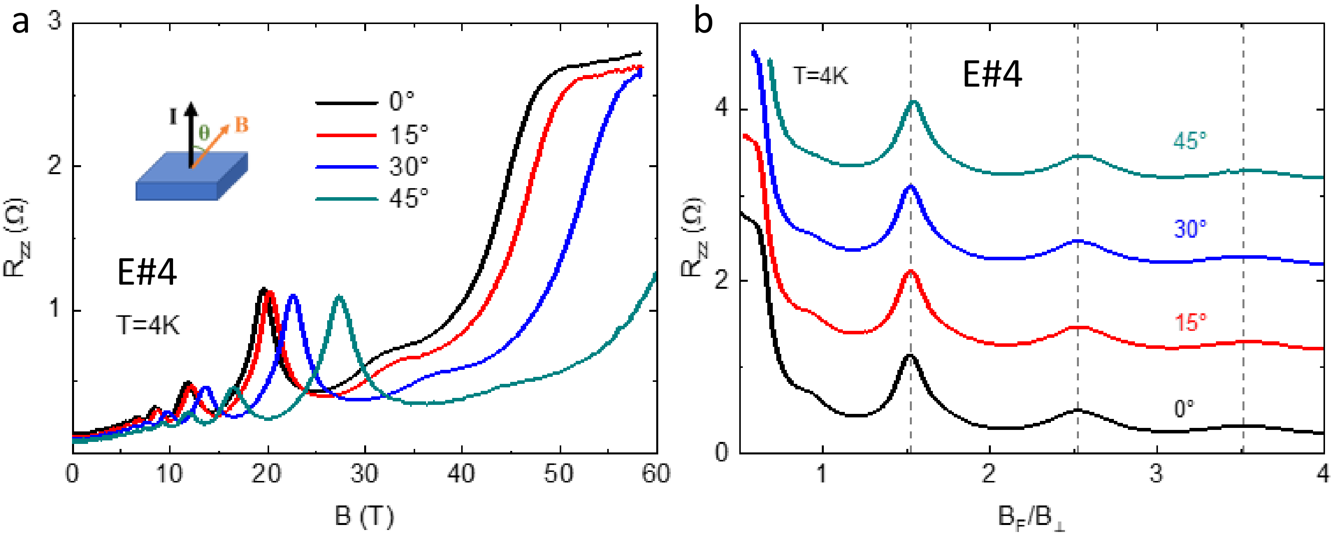

Supplementary Figure 12. (a) The out-of-plane resistance R_{zz} of a $Eu_{0.1}Ba_{0.9}MnSb_2$ sample (E#4) as a function of magnetic field under various field orientations. (b) R_{zz} vs. $B_F/B_\perp$ for sample E#4; B_F and $B_\perp$ refer to the SdH oscillation frequency and the perpendicular component of the magnetic field along the z-axis. The data in panel b are offset for clarity.

As shown in supplementary Fig. 12(b) where R_{ZZ} is plotted on the scale of $B_F/B_\perp$ (B_F, the SdH oscillation

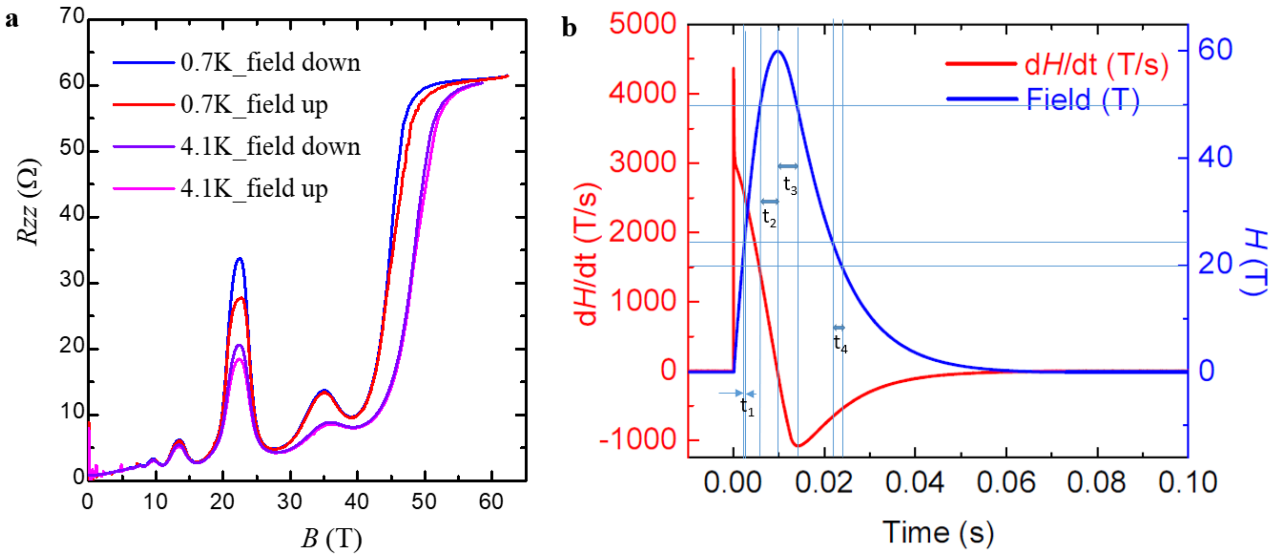

Supplementary Figure 13. **a**, The z-axis resistance R_{zz} measured in the up- (red/purple) and down-field (blue) sweeps for sample E#2 at 0.7 K and 4.1 K. **b**, The variation of pulse field with time and the derivative (red) of the field relative to time. $t_1(t_4)$ represents the time period for the field increasing (decreasing) from 20T (25T) to 25 T (20T). t_2 and t_3 represent the time periods of the field sweep from 50T to 60T and then from 60T to 50T respectively.

frequency; $B_\perp$, the perpendicular component of the magnetic field along the z-axis), we do not observe the expected spin-zero phenomena in $BaMnSb_2$, i.e. zero oscillation amplitude and phase inversion even when the tilt angle is increased to 45°. Instead, the oscillation amplitude and phase exhibit very small changes with the increase of θ, indicating that the g-factor of $BaMnSb_2$ is small, probably close to 2. Considering Sb is much lighter than Bi, it is reasonable to see $BaMnSb_2$ has a much smaller g factor than $EuMnBi_2$.

If we attributed the observed SdH oscillation peak splitting in ρ_{xx} (Fig. 4a in the main text) to the Zeeman effect, the g-factor would be estimated to ~12.6 using $F(\frac{1}{B_+} - \frac{1}{B_-}) = (\frac{1}{2})g(m_c/m_0)$ (F, oscillation frequency; $\frac{1}{B_+}$ and $\frac{1}{B_-}$ refer to the inverse fields of the split peaks) [16]. This is clearly not reasonable, since the g value of 12.6 should lead to the remarkable variation of oscillation amplitude and phase with the increase of θ, as seen in $EuMnBi_2$, which is not observed in our experiments. The indicates the observed oscillation peak splitting in ρ_{xx} or the valley splitting in ρ_{zz} should mostly originate from the orbital effect.

7. Possible origins of the z-axis resistance plateau in the quantum Hall state

The plateau in the z-axis resistance R_{zz} near 50T of sample E#2 (Fig. 4b) is a robust feature of the quantum Hall state within the quantum limit, that does not come from either joule heating or trivial surface states due to band bending as explained below. We can evaluate the heating effect by comparing the upward and downward field sweep measurements. supplementary Fig. 13b shows how the pulse field H varies with time t in upward and downward field sweeps as well as dH/dt (red curve). We present the comparison of R_{zz} measured in the up- and down-field sweeps in supplementary Fig. 13a, from which we can see the R_{zz} peak in the 20-25T range exhibits a striking difference between up- and down-field sweeps. In the main text, we have shown the $\gamma = 3/2$ ρ_{xy} plateau occurs within the 20-25T range (Fig. 4a and 4b). The difference of the R_{zz} peak height between the up- and down-field sweeps within this quantum Hall state should arise from heating effect. In the up-field sweep, the field increase from 20T to 25T takes an extremely short period of time t_1 (see supplementary Fig. 13b), such that the heat generated by the field sweep and measurements cannot be dissipated effectively. In contrast, the field decrease from 25T to 20T in the down-field sweep takes much longer time ($t_4 \approx 5t_1$, see supplementary Fig. 13b) so that the heating effect can be suppressed, which explains the enhanced R_{zz} peak probed in down-field sweep. However, the heating effect for the quantum Hall state within the quantum limit ($B > 50$T) becomes much weaker, because the R_{zz} probed above 50T shows much smaller

difference between up- and down-field sweeps (supplementary Fig. 13a). The time for the field increasing from 50T to 60T (t_2) and the time for the field decreasing from 60T to 50T (t_3) are much longer than t_1 and t_4 (see supplementary Fig. 13b), thus the heat generated by magnetic field sweeps and measurements within these time periods is expected to be small. Furthermore, we did similar up- and down-sweep measurements at 4.1 K and found the hysteresis of R_{zz} due to the heating effect is significantly suppressed for the quantum Hall state at 20-25T, and extremely small for the quantum Hall state near 50T. More importantly, the R_{zz} values at 4.1 K and 0.7K are nearly identical for fields close to 60T. If the R_{zz} plateau was due to heating effect, we would expect the R_{zz} value near 60T to decrease as the temperature increases to 4.1K, inconsistent with the observation of nearly identical R_{zz} at 0.7 K and 4.1K for fields approaching 60T. The evolution trend of the R_{zz} plateau from 0.7K to 4.1K also implies that the R_{zz} plateau should become more flattened as the temperature is further decreased below 0.7K. All these facts indicate that the R_{zz} plateau at the quantum Hall state within the quantum limit should be intrinsic and implies the presence of 2D chiral surface state as discussed in the main text.

Given the Dirac cones near the X point is gapped and the gap magnitude is small (∼50 meV), one may wonder the R_{zz} plateau is associated with the surface accumulation layers due to band bending. Such a possibility can be excluded for the following reasons: If trivial accumulation layers existed in $BaMnSb_2$, they would be present at the top and bottom surfaces along the z-direction, thus not contributing to the z-axis transport. On the other hand, if we assume the z-axis transport was associated with the trivial surface state, the SdH oscillations seen in R_{zz} would not be coupled to the bulk quantum Hall state, which clearly contradicts our experimental observation of R_{zz} reaching a plateau at the ρ_{xy} plateaus. As discussed in the main text, the SdH oscillations of R_{zz} and ρ_{xx} have the nearly same oscillation frequency for the samples taken from the same batch and the carrier densities extracted from the Hall coefficient and quantum oscillation frequency are consistent. These facts further indicate the trivial surface states due to band bending are not involved in $BaMnSb_2$.

II. SUPPLEMENTARY DISCUSSIONS

Tight-Binding Models

We first construct the TB models for the Sb layer, and study the evolution of the band in comparison with the first-principles calculation.

The orbital projection given by the first principles calculation (supplementary Figs. 15, 16 and 17) shows that the main contributions to the band near the Fermi level (slightly below zero energy) are from p_x and p_y orbitals of Sb atoms on the conducting Sb layers as discussed in the main text. Since the inter-layer tunneling is negligible as shown by the small bandwidth along $\Gamma - Z$ near the Fermi energy in Fig. 2(a) of the main text, we can construct the TB model only for one of the two equivalent conducting Sb layers in the conventional cell without any inter-layer tunneling along z direction. In the chosen conducting Sb layer (Fig 1(b) of the main text or supplementary Fig. 14), the two Sb atoms in one unit cell, labeled as Sb1 and Sb2, have sub-lattice vectors $\boldsymbol{\tau}_1 = (x_1 a, 0, c/2)$ and $\boldsymbol{\tau}_2 = (x_2 a, b/2, c/2)$, respectively, where a, b, c are the lattice constants of the conventional cell in x, y, z direction and $x_{1,2} \in [0, 1)$ and the values of $x_{1,2}$ will be given for different symmetry groups ($I4/mmm$ or $I2mm$). Therefore, the bases of the TB model are $|\boldsymbol{R} + \boldsymbol{\tau}_i, \alpha, s\rangle$ with the lattice vector $\boldsymbol{R} = (l_x a, l_y b, l_z c)$ ($l_{x,y,z} \in \mathbb{Z}$), the sublattice index $i = 1, 2$, the orbital index $\alpha = p_x, p_y$ and the spin-z index $s =\uparrow, \downarrow$. We include the on-site term H_0, the nearest-neighboring (NN) hopping H_1 and the next-NN hopping H_2 in the TB model, *i.e.* $H_{TB} = H_0 + H_1 + H_2$. Specifically, H_0 reads

$$H_0 = \sum_{\boldsymbol{R},i} c^\dagger_{\boldsymbol{R}+\boldsymbol{\tau}_i} M_i c_{\boldsymbol{R}+\boldsymbol{\tau}_i} \ , \tag{4}$$

where $c^\dagger_{\boldsymbol{R}+\boldsymbol{\tau}_i} = (c^\dagger_{\boldsymbol{R}+\boldsymbol{\tau}_i,p_x,\uparrow}, c^\dagger_{\boldsymbol{R}+\boldsymbol{\tau}_i,p_x,\downarrow}, c^\dagger_{\boldsymbol{R}+\boldsymbol{\tau}_i,p_y,\uparrow}, c^\dagger_{\boldsymbol{R}+\boldsymbol{\tau}_i,p_y,\downarrow})$. H_1 reads

$$H_1 = \sum_{\boldsymbol{R}} \sum_{n=1}^{4} c^\dagger_{\boldsymbol{R}+\Delta\boldsymbol{R}_n+\boldsymbol{\tau}_2} T_n c_{\boldsymbol{R}+\boldsymbol{\tau}_1} + h.c. \ , \tag{5}$$

where $\Delta\boldsymbol{R}_1 = 0$, $\Delta\boldsymbol{R}_2 = (a, 0, 0)$, $\Delta\boldsymbol{R}_3 = (a, -b, 0)$ and $\Delta\boldsymbol{R}_4 = (0, -b, 0)$. H_2 reads

$$H_2 = \sum_{\boldsymbol{R},i} \sum_{n=x,y} c^\dagger_{\boldsymbol{R}+\Delta\boldsymbol{R}_n+\boldsymbol{\tau}_i} Q_{ni} c_{\boldsymbol{R}+\boldsymbol{\tau}_i} + h.c. \ , \tag{6}$$

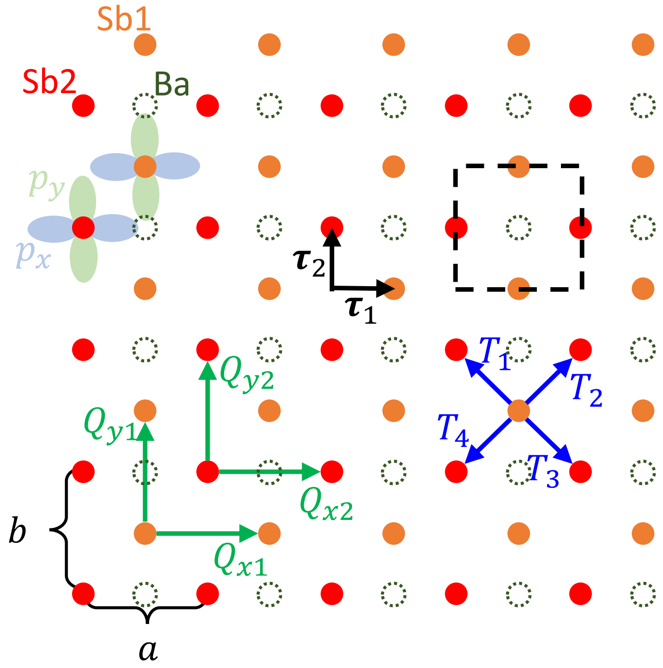

Supplementary Figure 14. This figure shows the positions of atoms, orbitals and the hopping. The red and orange atoms are Sb1 and Sb2, respectively, and the dark green dashed circles are the projection of nearby Ba atoms onto the Sb layer. The p_x and p_y orbitals of Sb atoms are labelled with light purple and light green colors, respectively. The black dashed line boxes the unit cell and the black arrows are the sublattice vectors $\boldsymbol{\tau}_1$ and $\boldsymbol{\tau}_2$. The blue and green arrows show the NN and next-NN hopping, respectively. a and b are lattice constants along x and y directions, respectively.

where $\Delta\boldsymbol{R}_x = (a, 0, 0)$ and $\Delta\boldsymbol{R}_y = (0, b, 0)$. (See more details about the hopping in supplementary Fig. 14.) Using $c^\dagger_{\boldsymbol{R}+\boldsymbol{\tau}_i} = \frac{1}{\sqrt{N'}}\sum_{\boldsymbol{k}} e^{-\mathrm{i}\boldsymbol{k}\cdot(\boldsymbol{R}+\boldsymbol{\tau}_i)} c^\dagger_{\boldsymbol{k},i}$ with N' the number of lattice cites and $c^\dagger_{\boldsymbol{k}} = (c^\dagger_{\boldsymbol{k},1}, c^\dagger_{\boldsymbol{k},2})$, we can transform H_{TB} to the momentum space, which reads

$$H_{TB} = \sum_{\boldsymbol{k}} c^\dagger_{\boldsymbol{k}} h(\boldsymbol{k}) c_{\boldsymbol{k}} \ , \tag{7}$$

where $h(\boldsymbol{k}) = h_0(\boldsymbol{k}) + h_1(\boldsymbol{k}) + h_2(\boldsymbol{k})$,

$$h_0(\boldsymbol{k}) = \begin{pmatrix} M_1 & \\ & M_2 \end{pmatrix} \ , \tag{8}$$

$$h_1(\boldsymbol{k}) = \begin{pmatrix} 0 & 0 \\ e^{-\mathrm{i}[k_x a(x_2-x_1)+k_y b/2]} & 0 \end{pmatrix} \otimes [T_1 + T_2 e^{-\mathrm{i}k_x a} + T_3 e^{-\mathrm{i}(k_x a - k_y b)} + T_4 e^{\mathrm{i}k_y b}] + h.c. \ , \tag{9}$$

$$h_2(\boldsymbol{k}) = \begin{pmatrix} Q_{x1} & \\ & Q_{x2} \end{pmatrix} e^{-\mathrm{i}k_x a} + \begin{pmatrix} Q_{y1} & \\ & Q_{y2} \end{pmatrix} e^{-\mathrm{i}k_y b} + h.c. \ . \tag{10}$$

As shown by the above equations, the TB model has no k_z dependence, coinciding with the fact that we do not include any inter-layer tunneling. Therefore, in the following, we only need to consider the $k_z = 0$ plane of the momentum space and redefine $\boldsymbol{k} = (k_x, k_y)$.

Before deriving the explicit forms of the terms in H_{TB}, let us first discuss the effect of anti-ferromagnetism (AFM). $BaMnSb_2$ has AFM with magnetic moments given by Mn atoms; such AFM structure can break some crystalline symmetries and the time-reversal (TR) symmetry. As discussed in the main text and above, the transport properties

of $BaMnSb_2$ are given by the conducting Sb layers, and the inter-layer tunneling, which passes through the Mn layer, is very small, implying the small effect of AFM on the transport. As shown in Fig. 2(a) of the main text, the negligible AFM effect is also reflected by the approximate 4-fold degeneracy near Fermi energy at X, since if the inter-layer tunneling and AFM are completely absent, the 4-fold degeneracy would become exact and come from the TR-protected Kramer's degeneracy in each of the two equivalent conducting Sb layers in one conventional cell. Therefore, we can construct the TB model without the AFM effect.

In the following, we first derive the forms of the terms in H_{TB} for the case where the system has no distortion and its space group is $I4/mmm$, and then consider the case where the zig-zag distortion reduces the space group to $I2mm$.

In this part, we first construct the TB model based on the symmetry property of the space group $I4/mmm$ and then compare the TB model with the first-principles calculations.

Owing to $I4/mmm$, we have $a = b$, and $(x_1, x_2) = (1/2, 0)$ for the chosen Sb layer. The generators of $I4/mmm$ are the four-fold rotation C_{4z} along z, two-fold rotation C_{2y} along y, inversion P and translation $T_{1/2}$ along $(a/2, b/2, c/2)$. As we neglect the AFM effect, we also have TR symmetry $\mathcal{T}$. $T_{1/2}$ relates the two conducting Sb layers in the conventional cell and just gives rise to the layer double degeneracy if neglecting the inter-layer tunneling. Therefore, we only need to consider C_{4z}, C_{2y}, P and $\mathcal{T}$ since we only construct the model for one conducting Sb layer in the conventional cell. The transformations of $c^\dagger_{\boldsymbol{R}+\boldsymbol{\tau}_i}$ under those symmetries are

$$\begin{aligned}
&C_{4z} c^\dagger_{\boldsymbol{R}+\boldsymbol{\tau}_i} C_{4z}^{-1} = c^\dagger_{C_{4z}(\boldsymbol{R}+\boldsymbol{\tau}_i)} C_{4z}^{OS} \\
&C_{2y} c^\dagger_{\boldsymbol{R}+\boldsymbol{\tau}_i} C_{2y}^{-1} = c^\dagger_{C_{2y}(\boldsymbol{R}+\boldsymbol{\tau}_i)} C_{2y}^{OS} \\
&P c^\dagger_{\boldsymbol{R}+\boldsymbol{\tau}_i} P^{-1} = c^\dagger_{-(\boldsymbol{R}+\boldsymbol{\tau}_i)} P^{OS} \\
&\mathcal{T} c^\dagger_{\boldsymbol{R}+\boldsymbol{\tau}_i} \mathcal{T}^{-1} = c^\dagger_{\boldsymbol{R}+\boldsymbol{\tau}_i} \mathcal{T}^{OS} \ ,
\end{aligned} \tag{11}$$

where $C_{4z}^{OS} = (-\mathrm{i}\tau_y) e^{-\mathrm{i}\frac{\sigma_z}{2}\frac{\pi}{2}}$, $C_{2y}^{OS} = \mathrm{i}\tau_z\sigma_y$, $P^{OS} = -\tau_0\sigma_0$, $\mathcal{T}^{OS} = \mathrm{i}\tau_0\sigma_y$, $\mathcal{T}$ is anti-linear, OS means the combination of orbital and spin, and τ's and σ's are Pauli matrices for orbital and spin indexes, respectively. Furthermore, the transformations of $c^\dagger_{\boldsymbol{k}}$ reads

$$\begin{aligned}
&C_{4z} c^\dagger_{\boldsymbol{k}} C_{4z}^{-1} = c^\dagger_{C_{4z}\boldsymbol{k}} \rho_x (-\mathrm{i}\tau_y) e^{-\mathrm{i}\frac{\sigma_z}{2}\frac{\pi}{2}} \\
&C_{2y} c^\dagger_{\boldsymbol{k}} C_{2y}^{-1} = c^\dagger_{C_{2y}\boldsymbol{k}} \mathrm{i}\rho_0\tau_z\sigma_y \\
&P c^\dagger_{\boldsymbol{k}} P^{-1} = c^\dagger_{-\boldsymbol{k}} (-\rho_0\tau_0\sigma_0) \\
&\mathcal{T} c^\dagger_{\boldsymbol{k}} \mathcal{T}^{-1} = c^\dagger_{-\boldsymbol{k}} \mathrm{i}\rho_0\tau_0\sigma_y \ ,
\end{aligned} \tag{12}$$

where ρ's are Pauli matrices for the sublattice index. The TB Hamiltonian H_{TB} must be invariant under C_{4z}, C_{2y}, P and $\mathcal{T}$, and thus we have the following forms for the terms in H_{TB} according to Eq. (11) (or equivalently Eq. (12)):

$$\begin{aligned}
&M_1 = \widetilde{m}_0\tau_0\sigma_0 + \widetilde{m}_1\tau_z\sigma_0 + \lambda_0\tau_y\sigma_z \ , \ M_2 = C_{4z}^{OS} M_1 (C_{4z}^{OS})^\dagger \ , \\
&T_1 = T_3 = t_0\tau_0\sigma_0 + t_1\tau_x\sigma_0 + it_2\tau_y\sigma_0 \ , \ T_4 = T_2 = \tau_z\sigma_y T_1 \tau_z\sigma_y \ , \\
&Q_{x1} = t_3\tau_0\sigma_0 + t_4\tau_z\sigma_0 \ , \ Q_{x2} = t_5\tau_0\sigma_0 + t_6\tau_z\sigma_0 \ , \ Q_{y1} = C_{4z}^{OS} Q_{x2} (C_{4z}^{OS})^\dagger \ , \ Q_{y2} = C_{4z}^{OS} Q_{x1} (C_{4z}^{OS})^\dagger \ ,
\end{aligned} \tag{13}$$

where all $\widetilde{m}$'s, t's and λ_0 are real and the spin-dependent hopping terms are neglected since they are typically high-order terms originating from the on-site SOC. Next, we analyze the physical meaning of each term in the above equation. $\widetilde{m}_{0,1}$ indicate the on-site energies, with $\widetilde{m}_0 + \widetilde{m}_1$ ($\widetilde{m}_0 - \widetilde{m}_1$) the on-site energy of p_x (p_y) orbital of Sb1. λ_0 is the on-site SOC. t_0 is the NN hopping between the same orbitals of different Sb atoms, *i.e.* from Sb1 p_x to Sb2 p_x or from Sb1 p_y to Sb2 p_y. $t_1 + t_2$ ($t_1 - t_2$) is the NN hopping from Sb1 p_y (p_x) to Sb2 p_x (p_y) for $\Delta\boldsymbol{R} = 0$. $t_3 + t_4$ ($t_3 - t_4$) is the next-NN hopping along x between p_x (p_y) orbitals of Sb1, while t_5 and t_6 have the same meaning for Sb2. We emphasize that the Ba atom in supplementary Fig. 14 is essential for the non-zero values of the parameters $\widetilde{m}_1$, t_2, $t_3 - t_5$ and $t_4 - t_6$. Without the effect of Ba atoms, we have $\widetilde{m}_1 = t_2 = t_3 - t_5 = t_4 - t_6 = 0$.

Now we discuss how the TB model qualitatively capture the main features of the first-principles calculation in the absence of SOC. We choose the following values of parameters:

$$\begin{aligned}
&\widetilde{m}_0 = 0 \ , \ \widetilde{m}_1 = 0.3\text{eV} \ , \ \lambda_0 = 0 \ , \ t_0 = 1\text{eV} \ , \ t_1 = 2\text{eV} \ , \ t_2 = 0 \ , \ t_3 = 0.1\text{eV} \ , \\
&t_4 = -0.06\text{eV} \ , \ t_5 = 0.15\text{eV and } t_6 = -0.06\text{eV} \ .
\end{aligned} \tag{14}$$

Here $\widetilde{m}_0 = 0$ means we set the average on-site energy to be the zero of energy, and $\lambda_0 = 0$ as we consider the case without SOC. The values of $\widetilde{m}_1$, t_2, $t_3 - t_5$ and $t_4 - t_6$ are close to zero since they are given by the high-order effects from Ba atoms, and we choose the NN hopping $t_{0,1}$ to have larger order of magnitudes than those of the next-NN hopping $t_{3,4,5,6}$. With Eq. (14), we plot the band structure and orbital projections of the TB model in supplementary

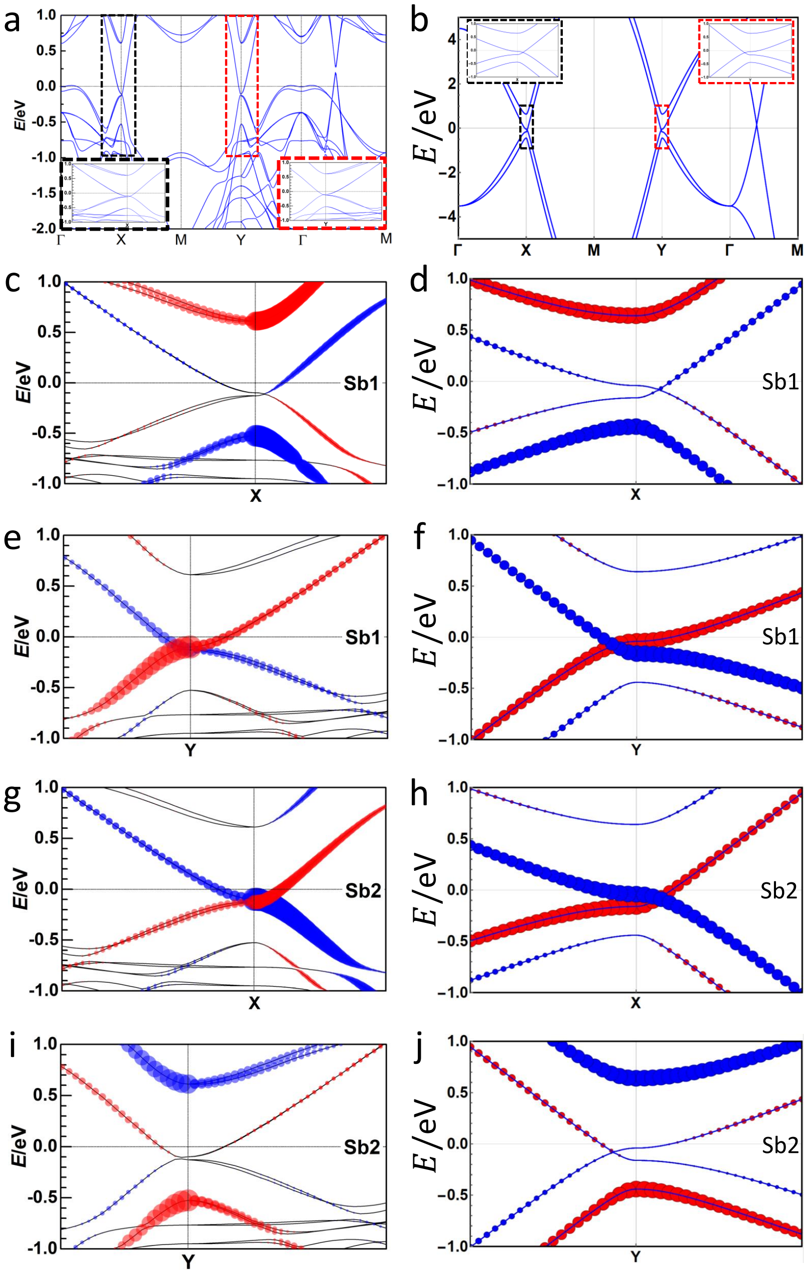

Supplementary Figure 15. (a), (c), (e), (g) and (i) are from the first-principles calculation for $I4/mmm$ without SOC, while the TB model (13) gives (b), (d), (f), (h) and (j) with parameter choice Eq. (14). (a) and (b) show the band structure with insets the zoom-in versions of the corresponding boxed parts in the main graphs. (c) and (d) show the orbital projection to Sb1 p_x and p_y orbitals at X. (e) and (f) show the orbital projection to Sb1 p_x and p_y orbitals at Y. (g) and (h) show the orbital projection to Sb2 p_x and p_y orbitals at X. (i) and (j) show the orbital projection to Sb2 p_x and p_y orbitals at Y. In all orbital projection plots, red (blue) bubbles stand for the p_x (p_y) orbital, and the diameters of bubbles are proportional to the projection values, *i.e.* larger diameter means larger contribution. For all plots around X (Y), the momentum axis is along $\Gamma - X - M$ ($M - Y - \Gamma$).

Fig. 15(b), (d), (f), (h) and (j). As shown in supplementary Fig. 15(a), (c), (e), (g) and (i), the key qualitative features of the the first principles calculation for $I4/mmm$ without SOC include (i) the gapless points along $X - M - Y$ formed by the crossing between p_x and p_y orbitals with opposite m_x and m_y parities , (ii) the large gap along $\Gamma - X$ and $Y - \Gamma$, (iii) Sb2 and Sb1 contribute to the bands closer to the Fermi energy at X and Y, respectively, and (iv) Sb2 p_y orbital has higher energy than Sb2 p_x at X. Here m_i means the mirror symmetry with mirror plane perpendicular to direction i. supplementary Fig. 15(b), (d), (f), (h) and (j) show that all the above qualitative features are captured by the TB model calculation for the I4/mmm case.

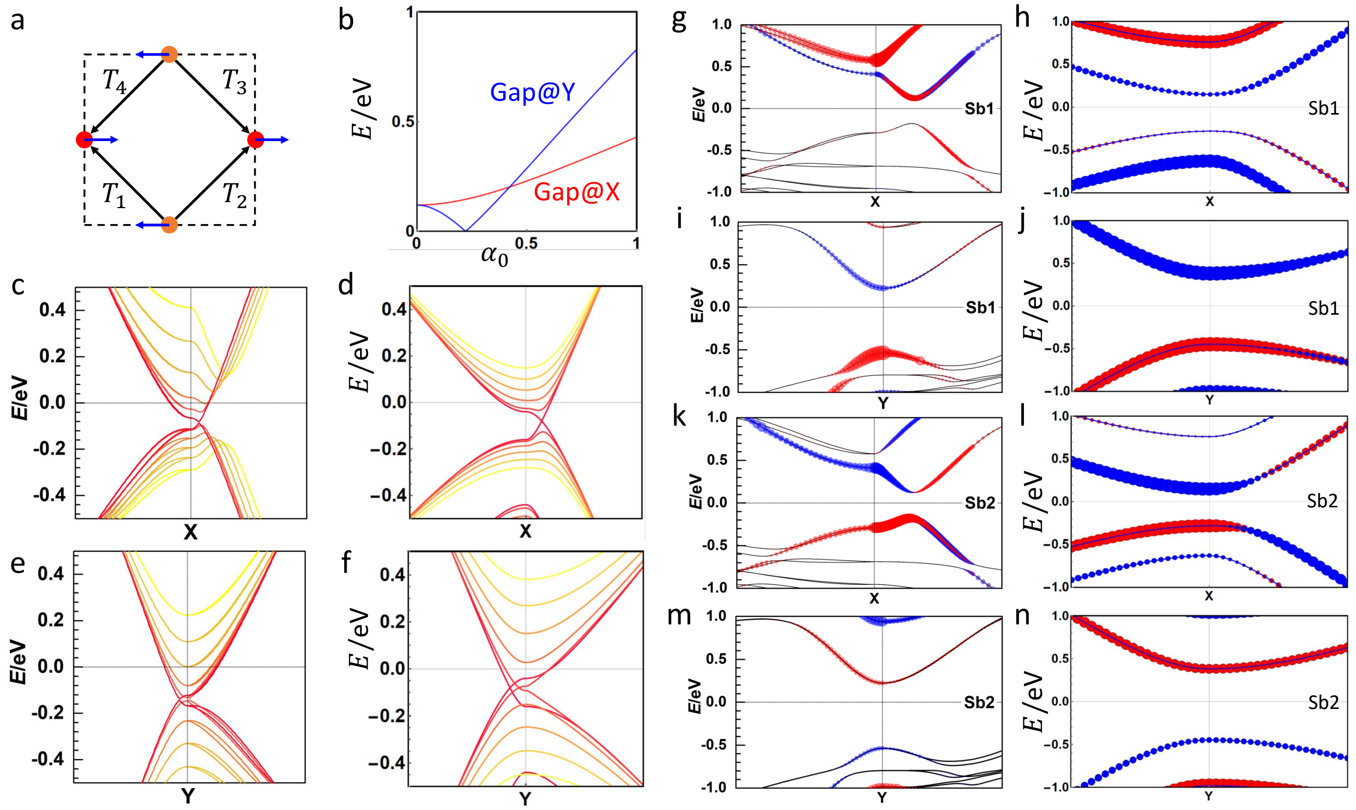

Supplementary Figure 16. Blue arrows in (a) show the relative shifts of atoms caused by the distortion in one unit cell (black dashed box). The black arrows are the NN hopping, and the red (orange) dot is Sb2 (Sb1) atom. (b) shows the change of the gap at X and Y as the distortion parameter α_0 increases, given by the distorted TB model (16) without SOC. (c) and (d) ((e) and (f)) show the evolution of band structure near X (Y) as distortion increases, where the distortion becomes larger from red to yellow. (g)-(n) show the orbital projection of Sb1 or Sb2 to bands near X or Y with p_x colored red and p_y colored blue. Here (c), (e), (g), (i), (k) and (m) are given by the first-principles calculation without SOC, while all others are given by the distorted TB model (16) without SOC. For all plots around X (Y), the momentum axis is along $\Gamma - X - M$ ($M - Y - \Gamma$).

In the next part, we add the zig-zag distortion to Eq. (13) to derive a TB model, and compare the model with the first-principles calculation with distortion.

The zig-zag distortion can be viewed as the shift of Sb1 atoms to the left and the shift of Sb2 atoms to the right, resulting in $a \neq b$, $x_1 = 0.4512$ and $x_2 = 0.01729$ as determined by the first-principles calculation. (See supplementary Fig. 16(a).) This distortion reduces the space group from $I4/mmm$ to $I2mm$ and preserves TR symmetry. The generators of $I2mm$ besides $T_{1/2}$ are the 2-fold rotation C_{2x} along x and the mirror operation m_y with mirror plane perpendicular to y. The symmetry transformations of $c^\dagger_{\boldsymbol{R}+\boldsymbol{\tau}_i}$ and $c^\dagger_{\boldsymbol{k}}$ under the two operations read

$$\begin{aligned}
C_{2x} c^\dagger_{\boldsymbol{R}+\boldsymbol{\tau}_i} C_{2x}^{-1} &= c^\dagger_{C_{2x}(\boldsymbol{R}+\boldsymbol{\tau}_i)} C_{2x}^{OS} \\
m_y c^\dagger_{\boldsymbol{R}+\boldsymbol{\tau}_i} m_y^{-1} &= c^\dagger_{m_y(\boldsymbol{R}+\boldsymbol{\tau}_i)} m_y^{OS} \\
C_{2x} c^\dagger_{\boldsymbol{k}} C_{2x}^{-1} &= c^\dagger_{C_{2x}\boldsymbol{k}} (-\mathrm{i}\rho_0\tau_z\sigma_x) \\
m_y c^\dagger_{\boldsymbol{k}} m_y^{-1} &= c^\dagger_{m_y\boldsymbol{k}} (-\mathrm{i}\rho_0\tau_z\sigma_y)\ ,
\end{aligned} \tag{15}$$

where $C_{2x}^{OS} = -\mathrm{i}\tau_z\sigma_x$, $m_y^{OS} = -\mathrm{i}\tau_z\sigma_y$ and TR transformation is shown in Eq. (11) and Eq. (12). In principle, one can use the above relations to derive all the symmetry allowed terms given by the distortion in H_{TB}, but this method is too complicated to capture the main physics. Instead, we define a distortion parameter α_0 with $\alpha_0 = 0$ and $\alpha_0 = 1$ corresponding to non-distorted and the fully-distorted cases, respectively, where the non-distorted case corresponds to the $I4/mmm$ case where $(x_1, x_2) = (\frac{1}{2}, 0)$ and the fully-distorted case corresponds to the realistic material with $(x_1, x_2) = (0.4512, 0.01729)$. Thus, we may include the distortion effect on the relative atom positions by choosing $x_1 = \frac{1}{2} + (0.4512 - \frac{1}{2})\alpha_0$ and $x_2 = 0.01729\alpha_0$. On the other hand, the distortion can change the hopping parameters, *e.g.* it can make $T_{1,4}$ larger than $T_{2,3}$ as shown in supplementary Fig. 16(a). For simplicity, we include such effect by

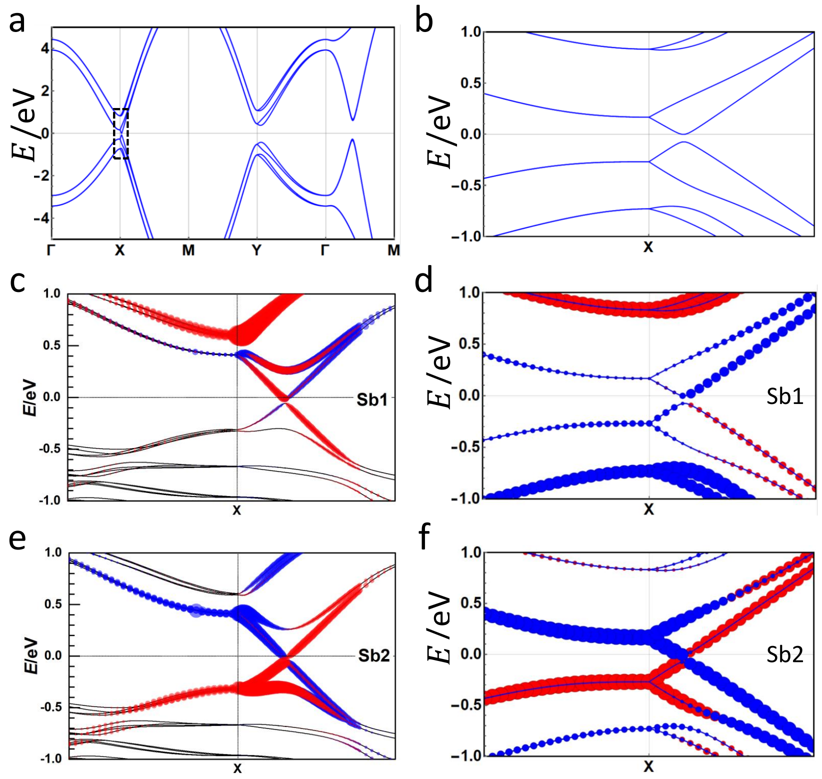

Supplementary Figure 17. (a) is the band structure given by the distorted TB model (16) with SOC, and (b) is the zoom-in version of the boxed part in (a). (c)-(f) show the orbital projection of Sb1 or Sb2 to bands near X with p_x colored red and p_y colored blue. Here (c) and (e) are given by the first-principles calculation with distortion and SOC, while (d) and (f) are given by the distorted TB model (16) with SOC. For all plots around X (Y), the momentum axis is along $\Gamma - X - M$ ($M - Y - \Gamma$).

revising Eq. (13) with

$$T_2 = \frac{\tau_z \sigma_y T_1 \tau_z \sigma_y}{f(\alpha_0)} \ , \ T_4 = \tau_z \sigma_y T_1 \tau_z \sigma_y \ , \ T_3 = \frac{T_1}{f(\alpha_0)} \ , \tag{16}$$

while keeping all other relations in Eq. (13) unchanged. It means that we simply make $T_{2,3}$ smaller than $T_{1,4}$ by a factor $1/f(\alpha_0) < 1$, while neglecting other changes of the on-site and hopping energy parameters in H_{TB} caused by the distortion. By fitting to the first-principles calculation, we get a simple linear form $f(\alpha_0) = 0.2\alpha_0 + 1$, and that Eq. (16) preserves $I2mm$ symmetry can be checked with Eq. (15).

Next, we compare the distorted TB model (16) with the first-principles calculation. We first focus on the case without SOC, and use the parameter values Eq. (14) to visualize the distortion effect from $\alpha_0 = 0$ to $\alpha_0 = 1$ with the distorted TB model (16). As shwon in supplementary Fig. 16(b), the gap at X increases as the distortion increases, while the gap at Y first closes and then reopens. In order to understand such feature, we show the evolution of the band structure near X and Y with the distortion in supplementary Fig. 16(d) and (f), in which the gapless points near X are directly gapped out as the m_x is broken by the distortion, while those near Y remain for small distortion since m_y is preserved. As the distortion increases, two gapless points near Y first move towards Y and then annihilate each other at Y, and finally a large gap at Y is opened in the fully-distorted case. Such evolution qualitatively matches the first-principles calculation for $I2mm$ without SOC as shown in supplementary Fig. 16(c) and (e). We further plot the orbital projection with the distorted TB model for $\alpha_0 = 1$ in supplementary Fig. 16(h), (j), (l) and (n) to compare with the first-principles calculation for the most energetically favorable distortion strength in supplementary Fig. 16(g), (i), (k) and (m), from which a qualitative match can be seen. In addition, according to supplementary Fig. 16, the anti-crossing between p_x and p_y is found along $X - M$. This anti-crossing originates from the gapless points along $X - M$ in $I4/mmm$ case, and the gap opening is induced by distortion in the I2mm case.

At last, in the fully-distorted case $\alpha_0 = 1$, we add the on-site SOC with $\lambda_0 = 0.25$eV to the distorted TB model (16), which removes the spin degeneracy and results in the formation of spin-split gapped Dirac cone as shown in supplementary Fig. 17(a) and (b), coinciding with Fig. 2(a) of the main text. The orbital projections are shown in supplementary Fig. 17(c) and (e) for the first-principles calculation and in supplementary Fig. 17(d) and (f) for the

distorted TB model, which coincide qualitatively.

Effective Models

Next we construct the effective models around X and Y for $I4/mmm$, around X for $I2mm$, and the effective model around the two valleys $\boldsymbol{K}_{\pm}$. Before going into the details, let us first discuss the chosen bases. As shown in supplementary Figs. 15, 16 and 17, the main contribution to the four bands, including the spin index and per conducting Sb layer, close to the X (Y) is from the p_x and p_y orbitals of Sb2 (Sb1). Therefore, the bases should be chosen as $c^\dagger_{\boldsymbol{k}_X+\tilde{\boldsymbol{k}},2,\alpha,s}$ for X and $c^\dagger_{\boldsymbol{k}_Y+\tilde{\boldsymbol{k}},1,\alpha,s}$ for Y, where $\boldsymbol{k}_X = (\pi/a,0,0)$, $\boldsymbol{k}_Y = (0,\pi/b,0)$, $\tilde{\boldsymbol{k}} = \boldsymbol{k}-\boldsymbol{k}_X$ for X, and $\tilde{\boldsymbol{k}} = \boldsymbol{k}-\boldsymbol{k}_Y$ for Y. In the following, we adopt the labels $c^\dagger_{\boldsymbol{k}_X+\tilde{\boldsymbol{k}},2} = (c^\dagger_{\boldsymbol{k}_X+\tilde{\boldsymbol{k}},2,p_x,\uparrow}, c^\dagger_{\boldsymbol{k}_X+\tilde{\boldsymbol{k}},2,p_x,\downarrow}, c^\dagger_{\boldsymbol{k}_X+\tilde{\boldsymbol{k}},2,p_y,\uparrow}, c^\dagger_{\boldsymbol{k}_X+\tilde{\boldsymbol{k}},2,p_y,\downarrow})$ and $c^\dagger_{\boldsymbol{k}_Y+\tilde{\boldsymbol{k}},1} = (c^\dagger_{\boldsymbol{k}_Y+\tilde{\boldsymbol{k}},1,p_x,\uparrow}, c^\dagger_{\boldsymbol{k}_Y+\tilde{\boldsymbol{k}},1,p_x,\downarrow}, c^\dagger_{\boldsymbol{k}_Y+\tilde{\boldsymbol{k}},1,p_y,\uparrow}, c^\dagger_{\boldsymbol{k}_Y+\tilde{\boldsymbol{k}},1,p_y,\downarrow})$ for convenience.

As discussed above, we need to consider 4 symmetry operations: C_{4z}, C_{2y}, P and $\mathcal{T}$, where the first three span the point group D_{4h}. [17] Since C_{4z} relates X with Y, we need to construct the effective models around X and Y simultaneously with both $c^\dagger_{\boldsymbol{k}_X+\tilde{\boldsymbol{k}},2}$ and $c^\dagger_{\boldsymbol{k}_Y+\tilde{\boldsymbol{k}},1}$. According to Eq. (12), the transformations of $c^\dagger_{\boldsymbol{k}_X+\tilde{\boldsymbol{k}},2}$ and $c^\dagger_{\boldsymbol{k}_Y+\tilde{\boldsymbol{k}},1}$ under those symmetry operations read

$$
\begin{aligned}
&C_{4z}c^\dagger_{\boldsymbol{k}_X+\tilde{\boldsymbol{k}},2}C_{4z}^{-1} = c^\dagger_{\boldsymbol{k}_Y+C_{4z}\tilde{\boldsymbol{k}},1}C_{4z}^{OS}\ ,\ C_{4z}c^\dagger_{\boldsymbol{k}_Y+\tilde{\boldsymbol{k}},1}C_{4z}^{-1} = c^\dagger_{\boldsymbol{k}_X+C_{4z}\tilde{\boldsymbol{k}},2}C_{4z}^{OS}\ ,\\
&C_{2y}c^\dagger_{\boldsymbol{k}_X+\tilde{\boldsymbol{k}},2}C_{2y}^{-1} = c^\dagger_{\boldsymbol{k}_X+C_{2y}\tilde{\boldsymbol{k}},2}C_{2y}^{OS}\ ,\ C_{2y}c^\dagger_{\boldsymbol{k}_Y+\tilde{\boldsymbol{k}},1}C_{2y}^{-1} = c^\dagger_{\boldsymbol{k}_Y+C_{2y}\tilde{\boldsymbol{k}},1}C_{2y}^{OS}\ ,\\
&Pc^\dagger_{\boldsymbol{k}_X+\tilde{\boldsymbol{k}},2}P^{-1} = c^\dagger_{\boldsymbol{k}_X-\tilde{\boldsymbol{k}},2}P^{OS}\ ,\ Pc^\dagger_{\boldsymbol{k}_Y+\tilde{\boldsymbol{k}},1}P^{-1} = c^\dagger_{\boldsymbol{k}_Y-\tilde{\boldsymbol{k}},1}P^{OS}\ ,\\
&\mathcal{T}c^\dagger_{\boldsymbol{k}_X+\tilde{\boldsymbol{k}},2}\mathcal{T}^{-1} = c^\dagger_{\boldsymbol{k}_X-\tilde{\boldsymbol{k}},2}\mathcal{T}^{OS}\ ,\ \mathcal{T}c^\dagger_{\boldsymbol{k}_Y+\tilde{\boldsymbol{k}},1}\mathcal{T}^{-1} = c^\dagger_{\boldsymbol{k}_Y-\tilde{\boldsymbol{k}},1}\mathcal{T}^{OS}\ ,
\end{aligned}
\tag{17}
$$

where the expressions of C_{4z}^{OS}, C_{2y}^{OS}, P^{OS} and $\mathcal{T}^{OS}$ can be found below Eq. (11). Next, we derive the form of the effective models around X and Y, *i.e.* $H_X = \sum_{\tilde{\boldsymbol{k}}} c^\dagger_{\boldsymbol{k}_X+\tilde{\boldsymbol{k}},2}h_X(\tilde{\boldsymbol{k}})c_{\boldsymbol{k}_X+\tilde{\boldsymbol{k}},2}$ and $H_Y = \sum_{\tilde{\boldsymbol{k}}} c^\dagger_{\boldsymbol{k}_Y+\tilde{\boldsymbol{k}},1}h_Y(\tilde{\boldsymbol{k}})c_{\boldsymbol{k}_Y+\tilde{\boldsymbol{k}},1}$, to the second order of $\tilde{\boldsymbol{k}}$ based on the symmetry properties listed in Eq. (17).

Since the effective models, $h_X(\tilde{\boldsymbol{k}})$ and $h_Y(\tilde{\boldsymbol{k}})$, consist of τ matrices, σ matrices and the momentum $\tilde{\boldsymbol{k}}$ to the second order, it is convenient to list their symmetry transformations according to Eq. (17), which are summarized in supplementary Tab. 3.

	τ_0	τ_x	τ_y	τ_z	σ_0	(σ_x,σ_y)	σ_z	$1,\ \tilde{k}_x^2+\tilde{k}_y^2$	$(\tilde{k}_x,\tilde{k}_y)$	$\tilde{k}_x\tilde{k}_y$	$\tilde{k}_x^2-\tilde{k}_y^2$
C_{4z}	+	−	+	−	+	$\begin{pmatrix} & -1 \\ 1 & \end{pmatrix}$	+	+	$\begin{pmatrix} & -1 \\ 1 & \end{pmatrix}$	−	−
C_{2y}	+	−	−	+	+	$\begin{pmatrix} -1 & \\ & 1 \end{pmatrix}$	−	+	$\begin{pmatrix} -1 & \\ & 1 \end{pmatrix}$	−	+
P	+	+	+	+	+	$\begin{pmatrix} 1 & \\ & 1 \end{pmatrix}$	+	+	$\begin{pmatrix} -1 & \\ & -1 \end{pmatrix}$	+	+
$\mathcal{T}$	+	+	−	+	+	$\begin{pmatrix} -1 & \\ & -1 \end{pmatrix}$	−	+	$\begin{pmatrix} -1 & \\ & -1 \end{pmatrix}$	+	+
IR	A_{1g}	B_{2g}	A_{2g}	B_{1g}	A_{1g}	E_g	A_{2g}	A_{1g}	E_u	B_{2g}	B_{1g}

Supplementary Table 3. The symmetry transformations of τ matrices, σ matrices and the momentum $\tilde{\boldsymbol{k}}$ to the second order under C_{4z}, C_{2y}, P and $\mathcal{T}$ operations. Here $\pm$ indicate their parities under the corresponding operation, and the transformation matrix, say R, follows the transformation rule $\boldsymbol{v}\to\boldsymbol{v}R$ for a row vector $\boldsymbol{v}$. The last row shows the irreducible representations (IRs) of D_{4h} according to the notation in Ref. [17].

From tensor products of elements in supplementary Tab. 3, we can construct the most general symmetry-allowed forms of $h_X(\tilde{\boldsymbol{k}})$ and $h_Y(\tilde{\boldsymbol{k}})$, which read

$$
\begin{aligned}
h_X(\tilde{\boldsymbol{k}}) &= (m_0 + B_{0x}\tilde{k}_x^2 + B_{0y}\tilde{k}_y^2)\tau_0\sigma_0 + (m + B_x\tilde{k}_x^2 + B_y\tilde{k}_y^2)\tau_z\sigma_0 + B_2\tilde{k}_x\tilde{k}_y\tau_x\sigma_0 + \lambda_0\tau_y\sigma_z\\
h_Y(\tilde{\boldsymbol{k}}) &= (m_0 + B_{0y}\tilde{k}_x^2 + B_{0x}\tilde{k}_y^2)\tau_0\sigma_0 + (-m - B_y\tilde{k}_x^2 - B_x\tilde{k}_y^2)\tau_z\sigma_0 + B_2\tilde{k}_x\tilde{k}_y\tau_x\sigma_0 + \lambda_0\tau_y\sigma_z\ .
\end{aligned}
\tag{18}
$$

Here we only include the on-site (thus $\tilde{\boldsymbol{k}}$-independent) SOC, *i.e.* the λ_0 term, and neglect third and higher orders of $\tilde{\boldsymbol{k}}$. To verify Eq. (18), we expand the TB model (13) around X and Y to the second order of the momentum, and project all terms other than SOC to the corresponding bases with second order perturbation, leading to the same form as Eq. (18) with the parameters given by

$$\begin{aligned}
&m_0 = \widetilde{m}_0 + 2t_3 - 2t_5,\ m = -\widetilde{m}_1 - 2(t_4 + t_6),\\
&B_{0x} = b^2\left(2t_0^2\left(\frac{1}{2(2t_3+t_4-2t_5+t_6)-\widetilde{m}_1} + \frac{1}{\widetilde{m}_1+4t_3-2(t_4+2t_5+t_6)}\right)+t_5\right),\\
&B_{0y} = b^2\left(-\frac{2(t_1-t_2)^2}{\widetilde{m}_1-2(2t_3+t_4-2t_5+t_6)} + \frac{2(t_1+t_2)^2}{\widetilde{m}_1+4t_3-2(t_4+2t_5+t_6)} - t_3\right),\\
&B_{x} = b^2\left(2t_0^2\left(\frac{1}{2(2t_3+t_4-2t_5+t_6)-\widetilde{m}_1} - \frac{1}{\widetilde{m}_1+4t_3-2(t_4+2t_5+t_6)}\right)+t_6\right),\\
&B_{y} = b^2\left(\frac{2(t_1-t_2)^2}{\widetilde{m}_1-2(2t_3+t_4-2t_5+t_6)} + \frac{2(t_1+t_2)^2}{\widetilde{m}_1+4t_3-2(t_4+2t_5+t_6)} + t_4\right),\\
&B_2 = -\frac{8b^2t_0(\widetilde{m}_1t_2+4t_1(t_5-t_3)-2t_2(t_4+t_6))}{(\widetilde{m}_1-2(2t_3+t_4-2t_5+t_6))(\widetilde{m}_1+4t_3-2(t_4+2t_5+t_6))}\,,
\end{aligned}\tag{19}$$

implying that all terms in Eq. (18) can naturally exist. As $a = b$ in this $I4/mmm$ case, all b's in the above relations can be replaced by a.

Before moving on to the case with distortion, let us discuss the signs of m, B_x, B_y in Eq. (18) according to the orbital projection shown in supplementary Fig. 15. Since supplementary Fig. 15 is done without SOC, we consider Eq. (18) without SOC, *i.e.* $\lambda_0 = 0$. At X, the energies of Sb2 p_x and p_y are $m_0 + m$ and $m_0 - m$, respectively, according to Eq. (18). Since the energy of Sb2 p_y is larger than that of Sb2 p_x at X as shown in supplementary Fig. 15, we have $m < 0$. Along $\Gamma - X$, the gap between Sb2 p_x and p_y bands reads $E_{p_x} - E_{p_y} = 2(m + B_x\tilde{k}_x^2)$ according to Eq. (18), and thus the large gap along $\Gamma - X$ in supplementary Fig. 15 gives $B_x < 0$. Along X-M, the gap between Sb2 p_x and p_y bands reads $E_{p_x} - E_{p_y} = 2(m + B_y\tilde{k}_y^2)$ according to Eq. (18), and thus the gapless point along X-M in supplementary Fig. 15 means $B_y > 0$. As a result, the positions of the two gapless points along X-M are

$$\boldsymbol{K}_\pm = (\pi/a, \pm\sqrt{-m/B_y}) \tag{20}$$

according to Eq. (18), corresponding to the two valleys defined in the main text.

As discussed above, the space group becomes $I2mm$ in the presence of the zig-zag distortion, and we only need to consider C_{2x}, m_y and $\mathcal{T}$ operations, where the first two span the point group C_{2v}. [17] As suggested by the first-principles and TB calculations (supplementary Figs. 16 and 17), we only need to consider the effective model around X with the bases $c^\dagger_{\boldsymbol{k}_X+\tilde{\boldsymbol{k}},2}$, which have the following transformations according to Eq. (15).

$$\begin{aligned}
&C_{2x}c^\dagger_{\boldsymbol{k}_X+\tilde{\boldsymbol{k}},2}C_{2x}^{-1} = c^\dagger_{\boldsymbol{k}_X+C_{2x}\tilde{\boldsymbol{k}},2}C_{2x}^{OS}\ ,\\
&m_yc^\dagger_{\boldsymbol{k}_X+\tilde{\boldsymbol{k}},2}m_y^{-1} = c^\dagger_{\boldsymbol{k}_X+m_y\tilde{\boldsymbol{k}},2}m_y^{OS}\ ,\\
&\mathcal{T}c^\dagger_{\boldsymbol{k}_X+\tilde{\boldsymbol{k}},2}\mathcal{T}^{-1} = c^\dagger_{\boldsymbol{k}_X-\tilde{\boldsymbol{k}},2}\mathcal{T}^{OS}\ ,
\end{aligned}\tag{21}$$

where C_{2x}^{OS}, m_y^{OS} and $\mathcal{T}^{OS}$ are listed below Eq. (15) and Eq. (11). Similar as supplementary Tab. 3, the matrices and momentum in $h_X(\tilde{\boldsymbol{k}})$ in this case can be classified based on their symmetry properties, as summarized in supplementary Tab. 4.

	τ_0	τ_x	τ_y	τ_z	σ_0	σ_x	σ_y	σ_z	1, $\tilde{k}_x^2$,$\tilde{k}_y^2$	$\tilde{k}_x$	$\tilde{k}_y$	$\tilde{k}_x\tilde{k}_y$
C_{2x}	+	−	−	+	+	+	−	−	+	+	−	−
m_y	+	−	−	+	+	−	+	−	+	+	−	−
$\mathcal{T}$	+	+	−	+	+	−	−	−	+	−	−	+
IR	A_1	B_1	B_1	A_1	A_1	A_2	B_2	B_1	A_1	A_1	B_1	B_1

Supplementary Table 4. The transformations of τ matrices, σ matrices and the momentum $\tilde{\boldsymbol{k}}$ to the second order under C_{2x}, m_y and $\mathcal{T}$ operations. Here ± indicate the parity under the corresponding operation. The last row shows the IRs of point group C_{2v} according to the notation in Ref. [17].

With on-site SOC and the spin-independent terms up to second order of momenta, the most general symmetry-allowed form of $h_X(\tilde{\boldsymbol{k}})$ for $I2mm$ can be derived from supplementary Tab. 4, and reads

$$h_X(\tilde{\boldsymbol{k}}) = (m_0 + B_{0x}\tilde{k}_x^2 + B_{0y}\tilde{k}_y^2)\tau_0\sigma_0 + (m + B_x\tilde{k}_x^2 + B_y\tilde{k}_y^2)\tau_z\sigma_0 + B_2\tilde{k}_x\tilde{k}_y\tau_x\sigma_0 + A_0\tilde{k}_y\tau_y\sigma_0 + \lambda_0\tau_y\sigma_z \ , \tag{22}$$

where A_0 is the extra term brought by the distortion as compared with Eq. (18). To check the above equation, we can re-derive the effective model (22) from Eq. (16) in the same way as above. As a result, the values of parameters in Eq. (22) can be determined by Eq. (14) and $\alpha_0 = 1$, which read

$$\begin{aligned}&m_0 = -0.0116764\text{eV}, m = -0.298474\text{eV}, B_{0x} = 2.88066a^2\text{eV}, B_{0y} = 10.5005b^2\text{eV}, B_x = -7.28705a^2\text{eV},\\ &B_y = 28.8769b^2\text{eV}, B_2 = -10.5988ab\text{eV}, A_0 = 5.24642b\text{eV} \ .\end{aligned} \tag{23}$$

At the end of this part, we discuss the effect of distortion based on Eq. (22) and supplementary Fig. 16. As supplementary Fig. 16 is done without SOC, we discuss Eq. (22) without SOC, *i.e.* $\lambda_0 = 0$. The larger Sb2 p_y energy indicates $m < 0$, and the large gap along $\Gamma - X$ shows $B_x < 0$. The band dispersion along $X - M$ is given by $m_0 + B_{0y}\tilde{k}_y^2 \pm \sqrt{(m + B_y\tilde{k}_y^2)^2 + (A_0\tilde{k}_y)^2}$ according to Eq. (22), and a clear band inversion signature along $X - M$ between Sb2 p_x and p_y is shown in supplementary Fig. 16(k) and (l). It indicates that $B_y > 0$, that gives rise to the gapless points at positions (20) for $A_0 = 0$. $A_0 \neq 0$ opens the gap and leads to the inverted band structure. Therefore, the distortion does not change the sign of m, B_x, B_y, and its main effect is introducing a non-zero A_0 to open the gap along $X - M$.

In this part, we try to capture the gapped Dirac cone feature in supplementary Fig. 17, which is plotted with distortion and SOC, by constructing the effective models around the two valleys. Now the bases should be chosen as $c^\dagger_{\boldsymbol{K}_\pm+\boldsymbol{q},2} = (c^\dagger_{\boldsymbol{K}_\pm+\boldsymbol{q},2,p_x,\uparrow}, c^\dagger_{\boldsymbol{K}_\pm+\boldsymbol{q},2,p_x,\downarrow}, c^\dagger_{\boldsymbol{K}_\pm+\boldsymbol{q},2,p_y,\uparrow}, c^\dagger_{\boldsymbol{K}_\pm+\boldsymbol{q},2,p_y,\downarrow})$, where $\boldsymbol{q} = \boldsymbol{k} - \boldsymbol{K}_\pm$, $\boldsymbol{K}_\pm = (\pi/a, \pm k_{y0})$ and $k_{y0} > 0$, and the effective Hamiltonian reads $H_{DC} = \sum_{\boldsymbol{q}}\sum_{\beta=\pm} c^\dagger_{\boldsymbol{K}_\beta+\boldsymbol{q},2} h_\beta(\boldsymbol{q}) c_{\boldsymbol{K}_\beta+\boldsymbol{q},2}$ with "DC" short for "Dirac cone". Although $k_{y0} = \sqrt{-m/B_y}$ according to Eq. (20) for the $I4/mmm$ case, in general it can be shifted by the distortion and terms with higher-order momenta. As the last part, we still only need to consider C_{2x}, m_y and $\mathcal{T}$ operations and the corresponding transformations according to Eq. (15) read

$$\begin{aligned}&C_{2x} c^\dagger_{\boldsymbol{K}_\beta+\boldsymbol{q},2} C_{2x}^{-1} = c^\dagger_{\boldsymbol{K}_{-\beta}+C_{2x}\boldsymbol{q},2} C_{2x}^{OS} \ ,\\ &m_y c^\dagger_{\boldsymbol{K}_\beta+\boldsymbol{q},2} m_y^{-1} = c^\dagger_{\boldsymbol{K}_{-\beta}+m_y\boldsymbol{q},2} m_y^{OS} \ ,\\ &\mathcal{T} c^\dagger_{\boldsymbol{K}_\beta+\boldsymbol{q},2} \mathcal{T}^{-1} = c^\dagger_{\boldsymbol{K}_{-\beta}-\boldsymbol{q},2} \mathcal{T}^{OS} \ ,\end{aligned} \tag{24}$$

where C_{2x}^{OS}, m_y^{OS} and $\mathcal{T}^{OS}$ are listed below Eq. (15) and Eq. (11). As the above transformations are similar as Eq. (21), the table of the symmetry properties of matrices and $\boldsymbol{q}$ in $h_\beta(\boldsymbol{q})$ is the same as supplementary Tab. 4 if replacing $\tilde{\boldsymbol{k}}$ by $\boldsymbol{q}$. As a result, the effective model around $\boldsymbol{K}_\pm$ to the leading order of $\boldsymbol{q}$ has the form

$$h_\pm(\boldsymbol{q}) = (E_0 \pm v_0 q_y)\tau_0\sigma_0 \pm v_2 q_y \tau_z\sigma_0 \pm v_1 q_x \tau_x\sigma_0 + (v_3 q_y \pm E_1)\tau_y\sigma_0 + \lambda_0\tau_y\sigma_z \ , \tag{25}$$

where we set the constant coefficient coupled to $\tau_z\sigma_0$ to zero by shifting the valley position, and we only include the on-site SOC that has the form $\tau_y\sigma_z$. The relation between Eq. (25) and Eq. (22) can be derived by choosing $\boldsymbol{K}_\pm$ as Eq. (20) and expanding Eq. (22) around $\boldsymbol{K}_\pm$ to the first order of $\boldsymbol{q}$, and reads

$$E_0 = -\frac{B_{0y}m - B_y m_0}{B_y}, E_1 = A_0\sqrt{-\frac{m}{B_y}}, v_0 = 2B_{0y}\sqrt{-\frac{m}{B_y}}, v_1 = B_2\sqrt{-\frac{m}{B_y}}, v_2 = 2B_y\sqrt{-\frac{m}{B_y}}, v_3 = A_0 \ . \tag{26}$$

Although the above relation cannot restrict the specific values of parameters in Eq. (25) since terms with higher order momenta in Eq. (22) may change the relation, it does indicate the main contribution and provides the following two points: (I) $v_2 < 0$ given by $B_y < 0$ discussed in the last part and (ii) E_1 and v_3 originate from the distortion as they are related with A_0. We next add a $\boldsymbol{q}$-quadratic term $\pm b_0(v_1^2 q_x^2 + v_2^2 q_y^2)\tau_y\sigma_0$ to Eq. (25) in order to explain the LL splitting in the next section, and Eq. (25) becomes

$$h_\pm(\boldsymbol{q}) = (E_0 \pm v_0 q_y)\tau_0\sigma_0 \pm v_2 q_y\tau_z\sigma_0 \pm v_1 q_x\tau_x\sigma_0 + [v_3 q_y \pm E_1 \pm b_0(v_1^2 q_x^2 + v_2^2 q_y^2)]\tau_y\sigma_0 + \lambda_0\tau_y\sigma_z \ . \tag{27}$$

We would like to emphasize that the $\boldsymbol{q}$-quadratic term that we add is just one of the symmetry-allowed $\boldsymbol{q}$-quadratic terms, and we neglect other terms since that one is enough to account for the LL splitting as discussed in the following and it simplifies the procedure of analytically solving the LL.

With $v_2 < 0$, we choose $\lambda_0 > 0$ and $E_1 < 0$ without loss of generality, and fit the Eq. (27) to the first-principles data, resulting in

$$E_0 = -0.0159125\text{eV}, E_1 = -0.12955\text{eV}, v_0 = -0.770856\text{ÅeV}, v_1 = 1.70245\text{ÅeV},$$
$$v_2 = 7.9056\text{ÅeV}, v_3 = -0.000840073\text{ÅeV}, \lambda_0 = 0.1531\text{eV}, b_0 = 1.5\text{eV}^{-1} . \quad (28)$$

The comparison between the fitted effective model and the first-principles data is shown in Fig. 18(b). As shown by Eq. (28), v_3 can be neglected, meaning that E_1 is the main effect of the distortion in Eq. (25).

At last, we discuss the spin-valley locking according to Eq. (27). From $h_\beta(0) = E_0\tau_0\sigma_0 + \beta E_1\tau_y\sigma_0 + \lambda_0\tau_y\sigma_z$, we know the eigenvalues of $h_\beta(0)$ are $E_0 + \beta E_1 + \lambda_0$, $E_0 + \beta E_1 - \lambda_0$, $E_0 - \beta E_1 - \lambda_0$ and $E_0 - \beta E_1 + \lambda_0$ with corresponding eigenstates $|p_+, \uparrow\rangle$, $|p_+, \downarrow\rangle$, $|p_-, \uparrow\rangle$ and $|p_-, \downarrow\rangle$, respectively. Here $p_\pm = (p_x \pm \mathrm{i}p_y)/\sqrt{2}$. The expressions of eigenvalues indicate that the appearance of SOC λ_0 in the presence of distortion E_1 always brings two bands closer with gap $2\,||E_1| - |\lambda_0||$ and takes the other two further apart with gap $2(|E_1| + |\lambda_0|)$. The gapped Dirac cone is naturally formed by the two closer bands as long as $|E_1| \neq |\lambda_0|$ with v_1 and v_2 terms in Eq. (27) giving the linear dispersion in the gapless case. Combing the eigenvalue expressions with the eigenstates, we find that the two bases that form the Dirac cone must have the same spin, and the spins at opposite valleys are opposite, resulting in the spin-valley locking. Such feature physically originates from the opposite on-site SOC for $p_\pm$, and thus the bands with the same spin always get pushed either closer or further apart by the SOC. As $E_1 < 0$ and $\lambda_0 > 0$ are chosen, the two bases that form the Dirac cone at $\boldsymbol{K}_+$ ($\boldsymbol{K}_-$) have spin up (down). It means that the spin up sector of h_+ and spin down sector of h_- in Eq. (25) account for the low-energy physics, which read

$$h_{+,\uparrow}(\boldsymbol{q}) = (E_0 + v_0 q_y)\tau_0 + v_2 q_y \tau_z + v_1 q_x \tau_x + (v_3 q_y + E_1 + b_0(v_1^2 q_x^2 + v_2^2 q_y^2))\tau_y + \lambda_0 \tau_y$$
$$h_{-,\downarrow}(\boldsymbol{q}) = (E_0 - v_0 q_y)\tau_0 - v_2 q_y \tau_z - v_1 q_x \tau_x + (v_3 q_y - E_1 - b_0(v_1^2 q_x^2 + v_2^2 q_y^2))\tau_y - \lambda_0 \tau_y \ . \quad (29)$$

Landau Levels

Next, we solve for the LLs of Eq. (29) in the presence of a uniform magnetic field along z, *i.e.* $\boldsymbol{B} = (0, 0, B)$. We denote the vector potential for the magnetic field as $\boldsymbol{A}$, *i.e.* $\boldsymbol{B} = \boldsymbol{\nabla} \times \boldsymbol{A}$.

We first discuss the modification of the models in Eq. (29) in order to solve for the LLs. The magnetic field can introduce the Zeeman term as $\mu_B B$ in $h_{+,\uparrow}(\boldsymbol{q})$ and as $-\mu_B B$ in $h_{-,\downarrow}(\boldsymbol{q})$ with μ_B the Bohr magneton, since the two Hamiltonians are in opposite spin sectors. As discussed in the main text, the Zeeman term is estimated to have smaller order of magnitude than the $\boldsymbol{q}$-quadratic term. We estimate the order of magnitude of the $\boldsymbol{q}$-quadratic term as follows. As the coefficients of the $\boldsymbol{q}$-quadratic term are $b_0 v_1^2$ and $b_0 v_2^2$, we may take the average of them $\kappa = \sqrt{(b_0 v_1^2)(b_0 v_2^2)}$ as their order of magnitude, and then the energy scale of the contribution of $\boldsymbol{q}$-quadratic terms to LLs can be estimated by $\kappa/l_B^2 \sim 0.3\text{meV}(B/\text{Tesla})$, which is much larger than the order of Zeeman term as discussed in the main text. Here $l_B = \sqrt{\hbar/(eB)}$ is the magnetic length and $e > 0$ is the elementary charge. The magnetic field also introduces the Peierls substitution $\boldsymbol{q} \to \boldsymbol{q} + e\boldsymbol{A}/\hbar$ to both $h_{+,\uparrow}(\boldsymbol{q})$ and $h_{-,\downarrow}(\boldsymbol{q})$ as the orbital effect. In addition, we want to transformation the bases from $p_{x,y}$ to $p_\pm$ with the unitary transformation

$$U = \begin{pmatrix} \frac{1}{\sqrt{2}} & \frac{1}{\sqrt{2}} \\ \frac{i}{\sqrt{2}} & -\frac{i}{\sqrt{2}} \end{pmatrix} . \quad (30)$$

With all the above three modifications, $h_{+,\uparrow}(\boldsymbol{q})$ turns into

$$\widetilde{h}_{+,\uparrow} = h_0 + h_1 + h_2 \ ,$$
$$h_0 = (E_0 + \mu_B B)\tau_0 + \text{sgn}(v_2) D_y \tau_x + \text{sgn}(v_1) D_x \tau_y,$$
$$h_1 = [E_1 + \lambda_0 + b_0(D_x^2 + D_y^2)]\tau_z \ ,$$
$$h_2 = \frac{v_0}{|v_2|} D_y \tau_0 + \frac{v_3}{|v_2|} D_y \tau_z \ , \quad (31)$$

where τ matrices are for $p_\pm$ now, $D_x = |v_1|(q_x + eA_x/\hbar)$ and $D_y = |v_2|(q_y + eA_y/\hbar)$. The modified $h_{-,\downarrow}$, noted as $\widetilde{h}_{-,\downarrow}$, can be derived by flipping the sign of $v_0, v_1, E_1, v_2, \lambda_0, b_0, \mu_B$ in $\widetilde{h}_{+,\uparrow}$, and thus we solve for the LLs of $\widetilde{h}_{+,\uparrow}$ in the following and derive the LLs of $\widetilde{h}_{-,\downarrow}$ by the sign flipping.

To solve for the LLs of $\widetilde{h}_{+,\uparrow}$, we first address h_0, and then take h_1 and h_2 into account by representing them with eigenstates of h_0. As suggested by Eq. (28), we choose $v_1 > 0$ and $v_2 > 0$. Since $[D_x, D_y] = -\mathrm{i}|v_1 v_2| eB/\hbar$, we can

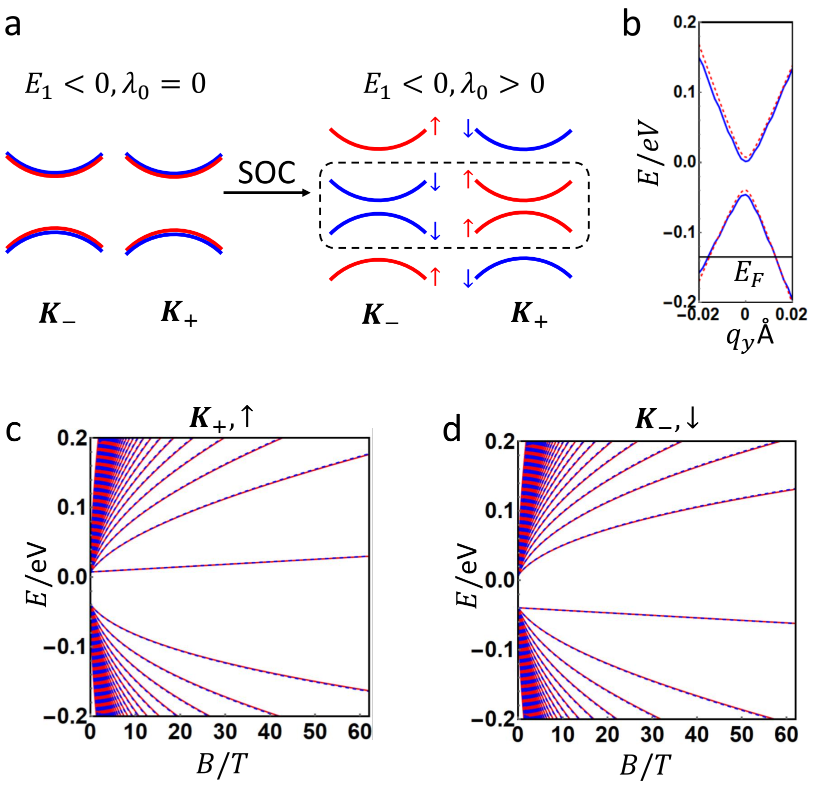

Supplementary Figure 18. (a) schematically shows the spin-valley locking of the gapped Dirac cone for $E_1 < 0$ and $\lambda_0 \geq 0$. The blue (red) bands have up (down) spin. The bands that form the gapped Direc cone are boxed by the black dashed line. (b) is the comparison of the band structures from the first-principles calculation (blue) and the effective model (red dashed) around K_+. The black line shows E_F. (c) and (d) compare the LLs (red solid lines) calculated numerically from $\widetilde{h}_{+,\uparrow}$ and $\widetilde{h}_{-,\downarrow}$, and the LLs (blue dashed lines) given by Eq. (35) and Eq. (36). (c) and (d) correspond to $\widetilde{h}_{+,\uparrow}$ and $\widetilde{h}_{-,\downarrow}$, respectively.

define $\hat{a} = (D_x - iD_y)\frac{l_B}{\sqrt{2|v_1 v_2|}}$, and have $[\hat{a}, \hat{a}^\dagger] = 1$. As a result, h_0 can be re-written as

$$h_0 = (E_0 + \mu_B B)\tau_0 + \frac{\sqrt{2|v_1 v_2|}}{l_B}\mathrm{i}\begin{pmatrix} 0 & -\hat{a}^\dagger \\ \hat{a} & 0 \end{pmatrix} , \tag{32}$$

and its eigenvalues and eigenstates read

$$h_0|\psi_0\rangle = (E_0 + \mu_B B)|\psi_0\rangle \ , \ h_0|\psi_l^\pm\rangle = \left(E_0 + \mu_B B \pm \frac{\sqrt{2|v_1 v_2| l}}{l_B}\right)|\psi_l^\pm\rangle \ , \tag{33}$$

where $|\psi_0\rangle = (|0\rangle, 0)^T$, $l = 1, 2, 3, ...$, $|\psi_l^\pm\rangle = (|l\rangle, \pm\mathrm{i}|l-1\rangle)^T/\sqrt{2}$, $\hat{a}^\dagger\hat{a}|0\rangle = 0$ and $\hat{a}^\dagger\hat{a}|l\rangle = l|l\rangle$.

Now we include h_1 and h_2. Although each LL of h_0 has degeneracy $1/(2\pi l_B^2)$, h_1 and h_2 do not break such degeneracy since they only depend on $\hat{a}, \hat{a}^\dagger$, and thus we can only focus on one degenerate subspace or equivalently omit the degenerate index. Within $\{|\phi_0\rangle, |\phi_l^\pm\rangle\}$ bases, the matrix element of h_1 has the form

$$[h_1]_{00} = E_1 + \lambda_0 + \frac{b_0|v_1 v_2|}{l_B^2} \ , \ [h_1]_{l'\gamma', l\gamma} = \left(E_1 + \lambda_0 + \frac{b_0|v_1 v_2|2l}{l_B^2}\right)\delta_{l'l}\delta_{\gamma',-\gamma} + \frac{b_0|v_1 v_2|}{l_B^2}\delta_{l'l}\delta_{\gamma',\gamma} \ , \ [h_1]_{l\gamma,0} = [h_1]_{0,l\gamma} = 0 \ , \tag{34}$$

where $\gamma, \gamma' = \pm$. On the other hand, h_2 couples LLs of h_0 with different energies since h_2 is linear in D_x and D_y. When the l is not too large, the inter-LL coupling given by h_2 is of order $\frac{\sqrt{|v_1 v_2|}}{l_B}\frac{v_3}{|v_2|}$ or $\frac{\sqrt{|v_1 v_2|}}{l_B}\frac{v_0}{|v_2|}$, while the LL spacing of h_0 is of order $\frac{\sqrt{|v_1 v_2|}}{l_B}$. According to Eq. (28), we have $|v_0/v_2| \sim |v_3/v_2| \ll 1$, suggesting h_2 can be neglected

for relatively low l. With this approximation, the LLs of $\widetilde{h}_{+,\uparrow}$ reads

$$\epsilon_0^{\boldsymbol{K}_+,\uparrow} = E_0+\mu_B B+E_1+\lambda_0+b_0\frac{|v_1v_2|}{l_B^2} \text{ and } \epsilon_{l,\pm}^{\boldsymbol{K}_+,\uparrow} = E_0+\mu_B B+b_0\frac{|v_1v_2|}{l_B^2}\pm\sqrt{\frac{2|v_1v_2|l}{l_B^2}+(E_1+\lambda_0+b_0\frac{|v_1v_2|}{l_B^2}2l)^2} \,. \quad (35)$$

By flipping the sign of $v_0, v_1, E_1, v_2, \lambda_0, b_0, \mu_B$ in the above equation, the LLs of $\widetilde{h}_{-,\uparrow}$ should have energies

$$\epsilon_0^{\boldsymbol{K}_-,\downarrow} = E_0-\mu_B B-E_1-\lambda_0-b_0\frac{|v_1v_2|}{l_B^2} \text{ and } \epsilon_{l,\pm}^{\boldsymbol{K}_-,\downarrow} = E_0-\mu_B B-b_0\frac{|v_1v_2|}{l_B^2}\pm\sqrt{\frac{2|v_1v_2|l}{l_B^2}+(-E_1-\lambda_0-b_0\frac{|v_1v_2|}{l_B^2}2l)^2} \,. \quad (36)$$

Eq. (35) and Eq. (36) together give the LLs relevent to the experiment, and it is clearly shown that the valley spltting is given by the Zeeman term μ_B and the $\boldsymbol{q}$-quadratic terms b_0. In order to verify the Eq. (35) and Eq. (36), we numerically solve the LLs with a cutoff on l: $l \leq 50$. The parameter choice is given by Eq. (28) and $b_0 = 1.5\text{eV}^{-1}$. During the procedure, we also represent the h_2 in $\{|\phi_0\rangle, |\phi_l^{\pm}\rangle\}$ bases using the fact that $[\hat{a}^\dagger\tau_0]_{l'\gamma',l\gamma} = \frac{\sqrt{l+1}+\gamma\gamma'\sqrt{l}}{2}\delta_{l',l+1}$, $[\hat{a}^\dagger\tau_0]_{1\gamma',0} = \frac{1}{\sqrt{2}}$, $[\hat{a}^\dagger\tau_z]_{l'\gamma',l\gamma} = \frac{\sqrt{l+1}-\gamma\gamma'\sqrt{l}}{2}\delta_{l',l+1}$,$[\hat{a}^\dagger\tau_z]_{1\gamma',0} = \frac{1}{\sqrt{2}}$ and the matrix elements of $\hat{a}^\dagger\tau_0$ and $\hat{a}^\dagger\tau_z$ are zero otherwise. As shown in supplementary Fig. 18, the match is quite good.

At the end of this part, we discuss the Shubnikov-de Haas oscillation in the presence of disorder. The disorder effect to the LLs can be included by introducing a Gaussian disorder broadening to the density of states (DOS):

$$DOS(E) = \frac{1}{2\pi l_B^2}\sum_n \frac{1}{\sqrt{\pi\Gamma^2}}\exp(-\frac{(E-E_n)^2}{\Gamma^2}) \,, \quad (37)$$

where $\Gamma = \Gamma_0\sqrt{B/\text{Tesla}}$, Γ_0 is the disorder broadening parameter, and n sums over all the LLs in Eq. (35) and Eq. (36). [18] From the above equation, we can determine the Fermi energy in presence of the disorder broadening by $\int_{E_0}^{E_F} DOS(E)dE = -n_0$, where the minus sign is due to the hole doping of the material. With the determined Fermi energy, we can then plot Fig. 4(f) of the main text with parameter choices Eq. (28), and $\Gamma_0 = 2$ or 3meV.

[1] J. Liu, J. Hu, H. Cao, Y. Zhu, A. Chuang, D. Graf, D. J. Adams, S. M. A. Radmanesh, L. Spinu, I. Chiorescu, and Z. Mao, Scientific Reports **6**, 30525 (2016).
[2] S. Huang, J. Kim, W. A. Shelton, E. W. Plummer, and R. Jin, Proceedings of the National Academy of Sciences **114**, 6256 (2017).
[3] M. A. Farhan, G. Lee, and J. H. Shim, Journal of Physics: Condensed Matter **26**, 042201 (2014).
[4] G. Cordier and H. Schäfer, Zeitschrift für Naturforschung B **32**, 383 (1977).
[5] M. Fiebig, V. V. Pavlov, and R. V. Pisarev, J. Opt. Soc. Am. B **22**, 96 (2005).
[6] S. A. Denev, T. T. A. Lummen, E. Barnes, A. Kumar, and V. Gopalan, Journal of the American Ceramic Society **94**, 2699 (2011), https://ceramics.onlinelibrary.wiley.com/doi/pdf/10.1111/j.1551-2916.2011.04740.x.
[7] S. Lei, M. Gu, D. Puggioni, G. Stone, J. Peng, J. Ge, Y. Wang, B. Wang, Y. Yuan, K. Wang, Z. Mao, J. M. Rondinelli, and V. Gopalan, Nano Letters **18**, 3088 (2018), pMID: 29631404.
[8] J. Liu, J. Hu, D. Graf, T. Zou, M. Zhu, Y. Shi, S. Che, S. Radmanesh, C. Lau, L. Spinu, *et al.*, Nature communications **8**, 646 (2017).
[9] A. Pippard, *The Dynamics of Conduction Electrons* (Gordon & Breach: New York, 1965).
[10] A. B. Pippard, *Magnetoresistance in metals*, Vol. 2 (Cambridge university press, 1989).
[11] D. Shoenberg, *Magnetic oscillations in metals* (Cambridge university press, 1984).
[12] M. V. Kartsovnik, Chemical Reviews **104**, 5737 (2004).
[13] J. Hu, Z. Tang, J. Liu, Y. Zhu, J. Wei, and Z. Mao, Phys. Rev. B **96**, 045127 (2017).
[14] M. V. Kartsovnik, Chemical Reviews **104**, 5737 (2004).
[15] H. Masuda, H. Sakai, M. Tokunaga, M. Ochi, H. Takahashi, K. Akiba, A. Miyake, K. Kuroki, Y. Tokura, and S. Ishiwata, Phys. Rev. B **98**, 161108 (2018).
[16] D. Shoenberg, *Magnetic oscillations in metals* (Cambridge university press, 2009).
[17] S. Altmann and P. Herzig, *Point-group theory tables* (Oxford, 1994).
[18] E. G. Novik, A. Pfeuffer-Jeschke, T. Jungwirth, V. Latussek, C. R. Becker, G. Landwehr, H. Buhmann, and L. W. Molenkamp, Phys. Rev. B **72**, 035321 (2005).
